# Whole Genome Mapping and Re-Organization of the Nuclear and Mitochondrial Genomes of *Babesia microti* Isolates

**DOI:** 10.1371/journal.pone.0072657

**Published:** 2013-09-04

**Authors:** Emmanuel Cornillot, Amina Dassouli, Aprajita Garg, Niseema Pachikara, Sylvie Randazzo, Delphine Depoix, Bernard Carcy, Stéphane Delbecq, Roger Frutos, Joana C. Silva, Richard Sutton, Peter J. Krause, Choukri Ben Mamoun

**Affiliations:** 1 Centre d'étude d'agents Pathogènes et Biotechnologies pour la Santé – UMR 5236, Montpellier, France; 2 Institut de Biologie Computationnelle, Montpellier, France; 3 Department of Internal Medicine, Section of Infectious Diseases, Yale School of Medicine, New Haven, Connecticut, United States of America; 4 Laboratoire de Biologie Cellulaire et Moléculaire (LBCM-EA4558), UFR Pharmacie, Université Montpellier 1, Montpellier, France; 5 Cirad, UMR 17, Cirad-Ird, TA-A17/G, Campus International de Baillarguet, Montpellier, France; 6 Institute for Genome Sciences and Department of Microbiology and Immunology, University of Maryland School of Medicine, Baltimore, Maryland, United States of America; 7 Yale School of Public Health and Yale School of Medicine, New Haven, Connecticut, United States of America; Johns Hopkins Bloomberg School of Public Health, United States of America

## Abstract

*Babesia microti* is the primary causative agent of human babesiosis, an emerging pathogen that causes a malaria-like illness with possible fatal outcome in immunocompromised patients. The genome sequence of the *B. microti* R1 strain was reported in 2012 and revealed a distinct evolutionary path for this pathogen relative to that of other apicomplexa. Lacking from the first genome assembly and initial molecular analyses was information about the terminal ends of each chromosome, and both the exact number of chromosomes in the nuclear genome and the organization of the mitochondrial genome remained ambiguous. We have now performed various molecular analyses to characterize the nuclear and mitochondrial genomes of the *B. microti* R1 and Gray strains and generated high-resolution Whole Genome maps. These analyses show that the genome of *B. microti* consists of four nuclear chromosomes and a linear mitochondrial genome present in four different structural types. Furthermore, Whole Genome mapping allowed resolution of the chromosomal ends, identification of areas of misassembly in the R1 genome, and genomic differences between the R1 and Gray strains, which occur primarily in the telomeric regions. These studies set the stage for a better understanding of the evolution and diversity of this important human pathogen.

## Introduction

Human babesiosis is an emerging tick-transmitted infection with a worldwide distribution that may cause prolonged or fatal disease primarily in neonates, adults over 50, those who acquire the infection through blood transfusion, and those who are asplenic, suffer from malignancy or HIV infection, or are immunodeficient for other reasons [1], [2]. Fatality rates of 6 to 9 percent have been reported among hospitalized patients and about 20 percent among those who are immunosuppressed or experience transfusion-transmitted babesiosis [1]–[4]. In the past several years, molecular techniques such as polymerase chain reaction (PCR) have been used to diagnose *B. microti* infections. However, *B. microti* presents several challenges to molecular investigation due to the lack of a long-term *in vitro* culture system and, until recently, of a genome assembly. Furthermore, the population diversity and associated virulence of *B. microti* pathogens are poorly characterized, properties that are best investigated through a comparative genomics approach. Hence, generation of the first genome sequence has remained a primary goal in the past few years.

The *Babesia microti* Genome Sequencing Project resulted in the first assembly, built from 140 Gb of raw sequence data, and representing ∼98% of the genome [5]. This assembly was arranged into three supercontigs corresponding to nuclear chromosomes, one mitochondrial genome and one apicoplast genome [5]. The initial release (under 4 accession numbers FO082868, FO082871, FO082872 and FO082874 corresponding to the nuclear chromosomes and the mitochondrial genome; the apicoplast genome has not yet been fully assembled) provided the first overview of the structure and composition of the genome of *B. microti.* Annotation of this genome revealed roughly 3500 predicted genes [5]. The genome of *B. microti* is the smallest among apicomplexan parasites sequenced to date, and about 28% the size of the *Plasmodium falciparum* genome [5]. This is of particular importance as both parasites invade and develop within human red blood cells, suggesting that understanding the intra-erythrocytic life cycle of *B. microti* may help define the minimal physiological requirements for successful development of hemoparasites within human erythrocytes. Unlike *Plasmodium*, no exo-erythrocytic life cycle has been reported for *B. microti*. Accordingly, the reconstruction of metabolic pathways from the annotated genome revealed a limited metabolic machinery and lack of enzymatic processes known to be important for *Plasmodium* development in hepatocytes [5]. In addition to these fundamental metabolic differences, phylogenetic analyses, which included a variety of protozoan species and more than 300 single copy genes, revealed that *B. microti* defines a new lineage among apicomplexan parasites distinct from the two families represented by *Babesia bovis* and *Theileria* species [5]. These results support the findings of previous studies based on 18S rRNA genes that suggested the placement of *B. microti* into a new lineage among piroplasms [6], [7], and revealed significant genome-wide and metabolic differences between *B. microti* and other piroplasma parasites [5].

The initial assembly of *B. microti* greatly advanced our understanding of the pathophysiology, metabolism and evolution of *B. microti*, and opened avenues for the development of better diagnostic tools and therapies [5]. However, several aspects of the genome of *B. microti* remained ambiguous, including the exact number of nuclear chromosomes, the structure of its mitochondrial genome and the exact size of the telomeric regions. These ambiguities were primarily due to the inability to adequately sequence and assemble telomeric regions, possible assembly errors due to the presence of highly repetitive and nearly identical sequence elements, and the difficulty of producing large amounts of mitochondrial DNA. Each of these problems can be addressed with additional approaches, including Whole Genome mapping technology (previously known as Optical Mapping) and detailed PCR and hybridization analyses. Whole Genome mapping reveals the architecture of complete genomes and has been used successfully in various areas of biology including comparative genomics, subtyping of human pathogens, epidemiology and forensics [8]–[12]. The technology has also been critical in closing the genome sequence of various human pathogens [8]–[12]. Unlike pulsed-field gel electrophoresis (PFGE) analyses, which can produce only a limited number of restriction fragments ordered by size on a gel, Whole Genome mapping involves digestion of the genomic DNA affixed to a glass substrate and allows precise mapping of contiguous fragments on each chromosome [13].

We used Whole Genome mapping and other molecular techniques to show that the genomes of two *B. microti* strains, R1 and Gray, each consists of four nuclear chromosomes and a linear mitochondrial genome. Whole Genome mapping provided further resolution of the length of each nuclear chromosome and 5′ and 3′ extremities. Comparison of the Whole Genome maps of the genomes of these two strains showed high conservation throughout the chromosomes except at the telomeric regions where major differences are present.

## Materials and Methods

### Ethics Statement

Animal protocol (CE-LR-0705) was reviewed and approved by the Institutional Animal Care and Use Committee of the University of Montpellier I.

### Purification of infected red blood cells and DNA extraction

The R1 and the Gray strains reported in this study were maintained in gerbils (*Meriones unguiculatus*). The R1 strain was isolated from an American patient during a visit to France [5]. It is unknown whether the strain originated in the USA or was contracted in France. The Gray strain is a standard laboratory strain and was isolated from Nantucket Island in 1970 [14]. These strains were used to determine whether similar nuclear and mitochondrial chromosomal structures are found in different *B. microti* strains and the extent of any genomic differences and alterations between them. Of particular importance is to establish whether the genome of a commonly used laboratory strain is representative of that of clinical isolates. Animal inoculation and housing were performed following approved protocol, CE-LR-0705 and according to national and international guidelines. Inoculations were made with 100 µl of blood from previously infected animals. Infected animals were monitored every three or four days for increased parasitemia by tail blood sampling following staining of thin blood smears using Giemsa stain. Once parasitemia reached 15–20%, animals were sacrificed and blood was collected by cardiac puncture with a heparinated syringe. Blood was immediately washed in 1X PBS. The Buffy coat was carefully discarded and white cells were removed by filtration on Plasmodipur columns (Europroxima, The Netherlands). Cell density was measured using a hemocytometer and parasitemia was determined by light microscopy of Giemsa-stained blood smears. Erythrocytes were used to prepare agarose plugs as described in [15] and 1 µg of *B. microti* DNA was used per plug. DNA for PCR and southern blot analyses was directly extracted from 100 µl of infected red blood cells using the Nucleospin® blood Quickpur extraction kit (Macherey-Nagel, Germany). The DNA concentration was measured using a BioSpecnano apparatus (Shimatzu, Japan).

### Pulsed-field gel electrophoresis (PFGE) analysis

Plugs were loaded on agarose gels and chromosomes were separated in 1X TAE or 0.5X TBE buffer. PFGE was performed using the CHEF procedure in a Gene Navigator™ system (GE healthcare life sciences). DNA digestion by NotI was validated after comparison of the restriction profile obtained with various enzymatic rare cutters. Plugs were washed twice in water and equilibrated twice in 1X NotI restriction buffer prior to enzymatic digestion. NotI digestion was performed on 1 µg of DNA at 37°C for 4 h in a 400 µl final volume solution containing 200 U of the enzyme. Size of the karyotype bands and NotI restriction fragments were evaluated using various standards: Mid Range I (from 15 to >250 kbp, New England Biolabs) and lambda concatemere (from 48.5 to >800 kbp, New England Biolabs), chromosomes from *Saccharomyces cerevisiae* (from 225 kbp to 1.9 Mbp, New England Biolabs), *Hansenula wingei* (from 1 to 3.1 Mbp, BioRad) and *Schizosaccharomyces pombe* (from 3.5 to 5.7 Mpb, Bio Rad). Gels were stained with ethidium bromide (0.8 µg/ml) for 45 min, de-stained in double-distilled water for 2 h and photographed under ultraviolet transillumination.

### PCR analyses and Southern blotting

PCR analyses were used to determine *B. microti* mitochondrial genome organization. These analyses were performed using the primers described in Table 1. PrimeSTAR HS DNA polymerase (Clontech-R010A) was used for PCR reactions. To determine the number of telomeric regions, Southern hybridizations were performed as previously described [15] using a ^32^P-labeled telomeric 28-mer oligonucleotide probe CCCTGAACCCTAAACCCTGAACCCTAAA. This sequence is 93% identical to the sequence found in the telomeric regions of the R1 isolate. Southern hybridization to evaluate the organization of the mitochondrial genome was performed following separation of total DNA on a 0.7% agarose gel and probing with a DIG-labeled specific probe. The probes were obtained following PCR amplification using the following specific primers: cox1-F: 5′- TTCATAGTTACCTTTACACTAGGTGGTAC, cox-R: 5′- CTATAGCGTTAAGATGTATAGTAATAGTATC; cob-F: 5′-GACACCATTACATATAGTACCTGAATGG, cob-R: 5′- CTATAGGGATCGTAGTCGTGTACTGC; cox3-F: 5′-TTGGATTAGGTTGTGCAGCAACAACAC, cox3-R: 5′-TTAATACATGTACAATAAGGATAATAGTGATAGCC. The probes were labeled using DIG PCR labeling kit (Roche applied science). Hybridization signals were detected by chemioluminescence using an anti-Digoxigenin-AP Fab fragment (Roche applied science) and CDP-Star reagent as substrate (New England Biolabs).

**Table 1:** Primers used to validate the linear configuration and 4 structural types of the mitochondrial genome.

Primers	Sequence
1	5′- AGA GGA GAG CTA GGT AGT AGT GGA G -3′
2	5′- CTC CAC TAC TAC CTA GCT CTC CTC T -3′
3	5′- CCA AAT GAG CTA CTG GGG AGC TAC -3′
4	5′- GGA AGT GAG CTA CCA CAT ACG CTG TA -3′
5	5′- GTA CAG ACA GTG GAG CAG AGG AAC -3′
6	5′- GTT CCT CTG CTC CAC TGT CTG TAC -3′
7	5′- TAA CAG TCT CTA CTC CTC TAC CCT G -3′
8	5′- TAG TGA TAG CCA TAC AGC TTC TAC G -3′
9	5′- CGT AGA AGC TGT ATG GCT ATC ACT A -3′

### Whole Genome Mapping

The Whole Genome Maps (WGM) of *Babesia microti* R1 and Gray strains were prepared by OpGen (Gaithersburg, MD USA), using a proprietary technique. Agarose plugs were prepared in different conditions. The plugs containing the infected erythrocytes were kept for two days at 50°C in a ESPK solution (EDTA 0.5M, SLS 0.5% and Proteinase K 2 mg/ml). The solution was renewed after one day. Plugs were then stored at 4°C. Low melting temperature agarose plug(s) were resuspended in 1X TE buffer and transferred to a sterile Petri dish. The plugs were prewarmed at 70°C and treated with Beta-agarase for 8h at 42°C. The DNA solution was resuspended in loading buffer and quantified using a QCard. Fragments over 150 kb of the genomic DNA were subsequently stretched and immobilized along microfluidic channels. The aligned fragments were then treated with KpnI, resulting in breaks within the fragment while the order of digested fragments is maintained. KpnI was chosen following detailed bioinformatics analyses aimed to select an enzyme capable of generating fragment sizes suitable for optical mapping. These fragments were stained with a fluorescent dye and the fluorescence intensity measured and analyzed to determine fragment size and to create Single Molecule Restriction Maps or SMRMs. Finally, these SMRMs are assembled by overlapping fragment patterns to produce a WGM. This map was then compared with the *in silico* restriction map inferred from the *B. microti* assembly available in GenBank. Detailed information about the technique was reported by Shukla et al. [16].

## Results

### The nuclear genome of *B. microti* consists of four chromosomes

**Figure 1:**
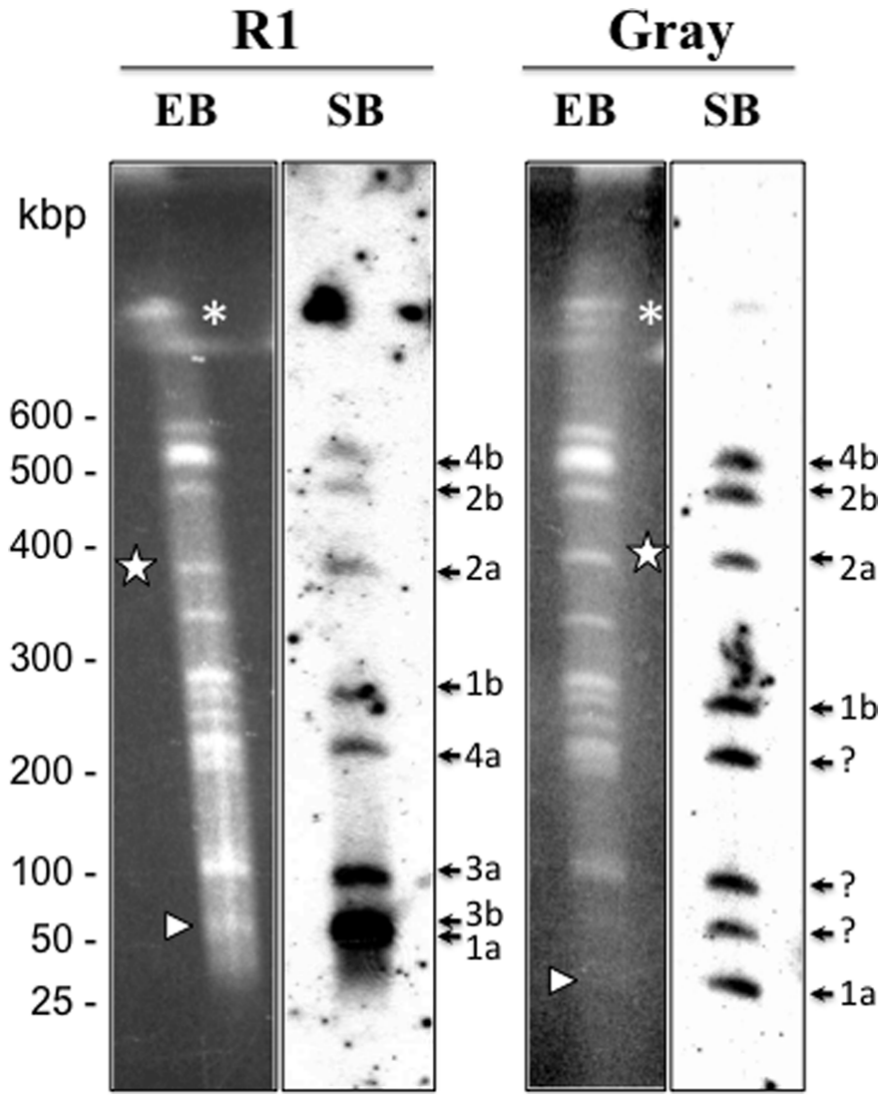
1D-PFGE *Not*I Restriction Fragment Length Polymorphism (RFLP) analyses of *B. microti* R1 and Gray strains (left) and their corresponding Southern hybridizations (right) using a radiolabeled telomeric probe. PFGE conditions used are: 1% agarose gel in 1X TAE using a pulse condition of 1 min for 10h at 6 V/cm (10.5°C). Arrows indicate the location of the 8 telomeric regions in the *B. microti* genome. Fragments showing restriction polymorphism are indicated with white arrows and stars. *: Undigested genomic DNA. 1: Chromosome I, 2: Chromosome II, 3: Chromosome III, 4: Chromosome IV. a and b represented the two telomeric regions of each chromosome.

Previous efforts to separate the chromosomes of two *B. microti* strains, R1 and Gray, by PFGE analyses, suggested the presence of three chromosomes: KI, KII and KIII [5]. However, further analysis of the intact and digested forms of the largest predicted chromosome, KIII, indicated that a three-chromosome nuclear genome model in *B. microti* might not be accurate. The presence of repetitive sequence elements in the telomeric and subtelomeric regions precluded a clear option between a three-chromosome model, in which one chromosome is large and more than twice the size of the other two, and a four-chromosomal model, in which the two largest chromosomes are roughly the same size, and just slightly larger than the two smallest chromosomes. To resolve this issue, chromosomes purified from the R1 and Gray strains were digested with the restriction enzyme NotI and the resulting fragments were separated by PFGE prior to hybridization with a radiolabeled probe conserved in the telomeric regions of each chromosome. Consistent with a four-chromosome genome, Southern hybridization revealed 8 bands corresponding to eight telomeric regions of four chromosomes (Fig. 1). Two of these bands (1a and 2a) correspond to the restriction fragments previously shown to display length polymorphism between the R1 and Gray strains [5].

**Figure 2:**
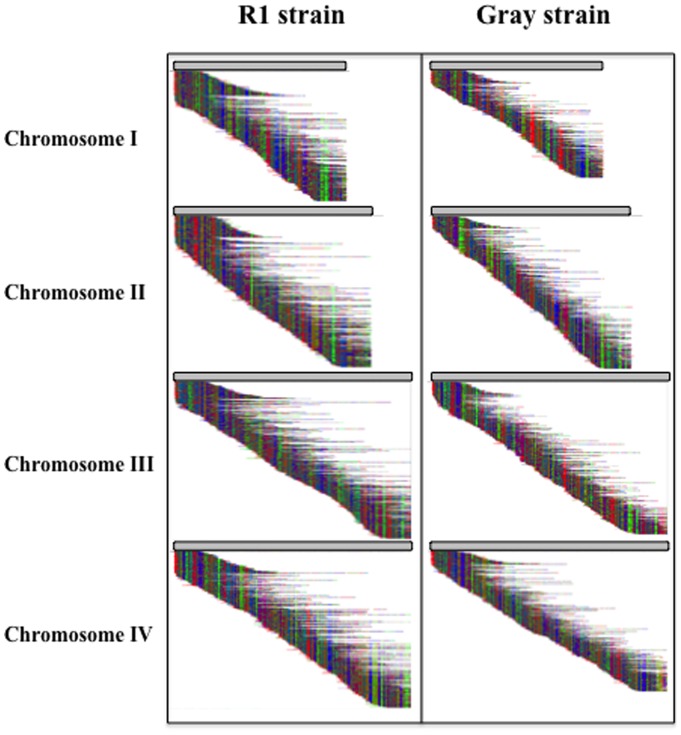
Whole Genome Map Assemblies of chromosomes I, II, III and IV of the *B. microti* R1 and Gray strains. Each multicolored horizontal line represents a single molecular map. Colors are used to distinguish KpnI restriction fragments. Blunt ends indicate true ends of chromosomes. The horizontal gray line on top of the assemblies represents the assembled chromosome from each map contig.

In order to further validate the presence of four chromosomes in the genome of *B. microti*, we have generated a Whole Genome map (WGM) of the entire genomes of the R1 and Gray strains. This approach has been successfully used to validate chromosome numbers in the Necrotrophic fungal pathogens Scle*rotinia sclerotiorum* and *Botrytis cinerea* [17] as well as the protozoan parasites *Plasmodium falciparum* and *Leishmania major* [18]–[20]. High molecular weight DNA was prepared embedded in Low Melting Temperature agarose plugs and further processed to obtain working DNA solutions. Following stretching and immobilization of the single DNA molecules along microfluidic channels, the molecules were digested with the restriction enzyme KpnI to create ordered fragments. Staining with a fluorescent dye and further assembly using MapSolver™ software resulted in WGMs consisting of 4 distinct assemblies corresponding to 4 chromosomes for each strain (Table 2). Blunt ends were observed at each contig ends indicating that the full length of each chromosome was resolved (Fig. 2). Assembly statistics indicated a minimum coverage of 37x and a maximum of 198x across all chromosomes with very large read lengths, strongly supporting a nuclear genome with 4 chromosomes, which is consistent with the PFGE data. Furthermore, DNA molecules providing coverage ranged on average between 351 kb (e.g. chromosome III of the Gray strain) and 468 kb (e.g. chromosome III of the R1 strain) with the largest molecule reaching 1,677 kb (e.g. chromosome II of the R1 strain, which represents almost the entire chromosome covered by a single molecule).

**Table 2:** Whole Genome Mapping assembly statistics for *Babesia microti* Gray and R1 strains.

*Babesia microti* (Gray strain)	Chromosome 1	Chromosome 2	Chromosome 3	Chromosome 4
Chromosome Size (Mb)	1.88	1.85	1.60	1.35
Minimum Coverage	37X	43X	45X	57X
Maximum Coverage	139X	140x	163X	153X
Minimum Molecule Size (kb)	155	165	165	159
Maximum Molecule Size (kb)	992	999	902	953
Average Molecule Size (kb)	356	370	351	363
***Babesia microti*** ** (R1 strain)**	**Chromosome 1**	**Chromosome 2**	**Chromosome 3**	**Chromosome 4**
Chromosome Size (Mb)	1.88	1.85	1.58	1.38
Minimum Coverage	91X	75X	77X	88X
Maximum Coverage	175X	189X	198X	195X
Minimum Molecule Size (kb)	165	170	166	168
Maximum Molecule Size (kb)	1558	1677	1504	1180
Average Molecule Size (kb)	453	441	468	465

**Figure 3:**
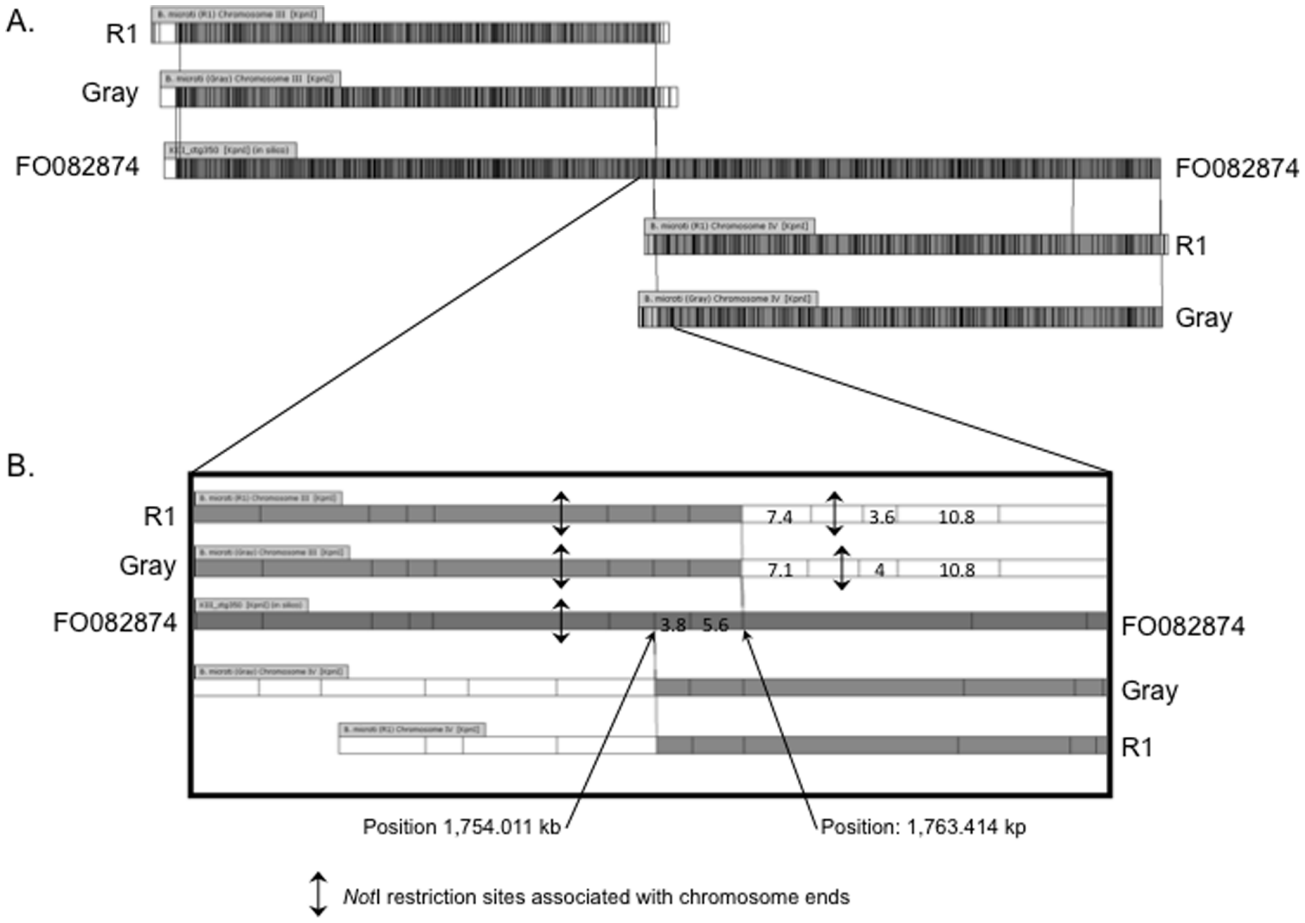
Comparison of the middle region of the KpnI restriction map of contig KIII_ctg350 with the 3′ end and 5′ end of chromosomes III and IV, respectively. The 3′ end of chromosome III (IIIb) and 5′ end of chromosome IV (IVa) share a common region, which includes two KpnI fragments of 3.8 and 5.6 kb. This region is in one orientation on chromosome III and the opposite orientation on chromosome IV. The regions of the chromosomes missing from the original assembly are shown in white (sizes are in kb).

**Figure 4:**
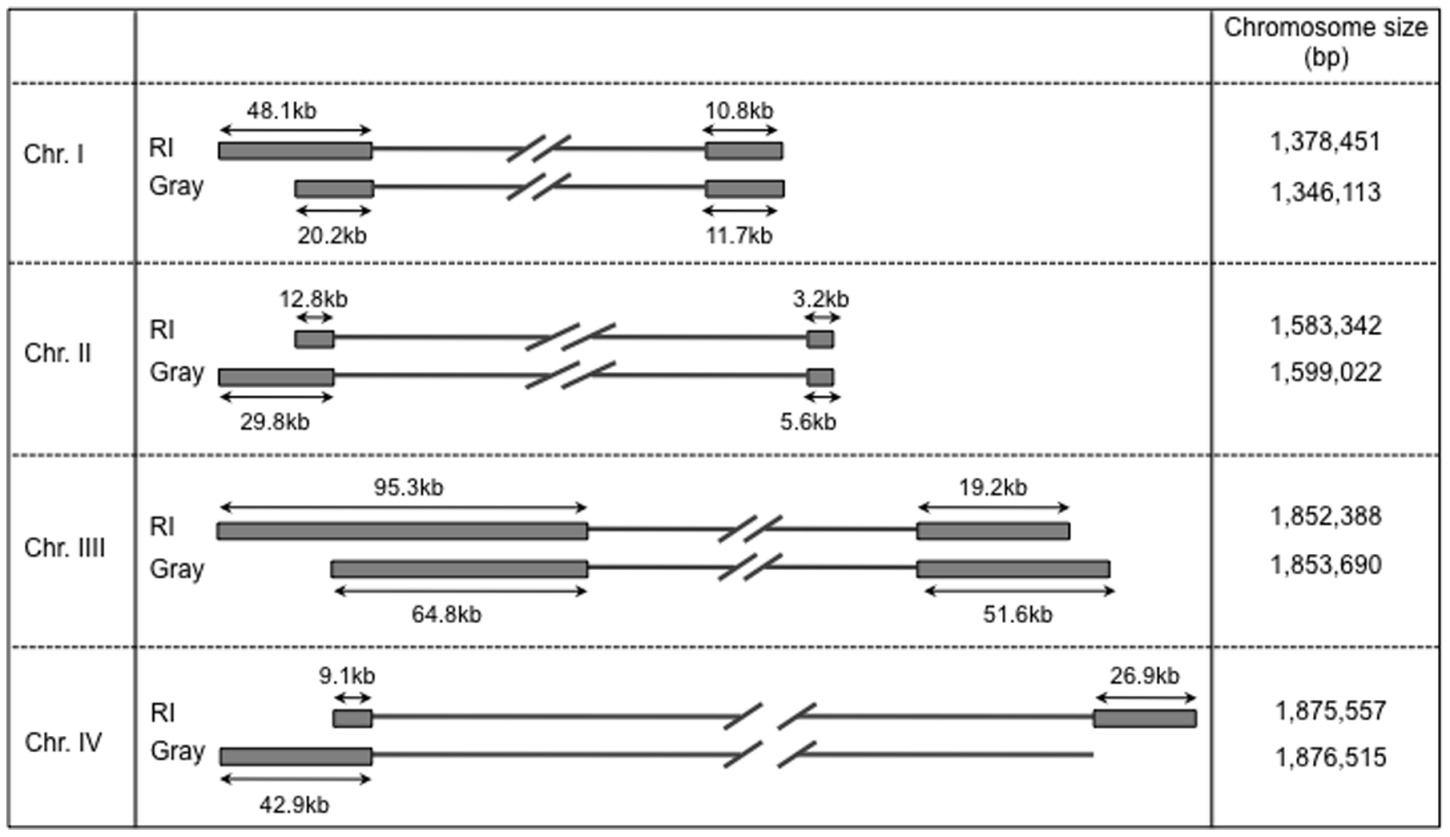
Schematic representation of the extremities of the four chromosomes of the R1 and Gray strains based on WGM analysis. Gray boxes represent the terminal ends of each chromosome as revealed by Whole Genome Mapping. Chromosome sizes are based on WGM calculations.

Chromosomes III and IV each align to one half of the previously reported contig KIII_ctg350 (former database entry FO082874) and share a highly conserved 9.4 kb region close to the beginning of one of their telomeric regions (Fig. 3). Sequence analysis shows that this fragment contains members of previously described multigene families (IIIc region described in [5]). The presence of this repeated region and the inability to obtain full sequences of the telomeric regions were in large part responsible for the merging of chromosomes III and IV into a single supercontig [5]. Whole Genome mapping further showed that these two chromosomes differ in size by only ∼23 kb (Fig. 4 and Table S1 in Supporting Information S1). This explains why the two chromosomes could not be separated using standard PFGE chromosomal separation techniques.

**Figure 5:**
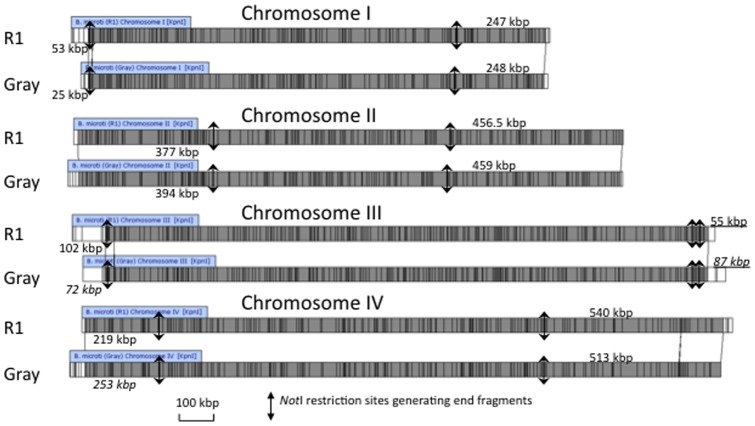
Comparison of the Whole Genome maps (WGM) of each chromosome of the R1 and Gray strains. Restriction sites are shown as vertical lines within each WGM. Differences between maps are indicated by the alignment connectors. The regions of the chromosomes that are different between the two WGMs are shown in white. The NotI restricted site and predicted size of the restriction fragments are represented for each extremity. Underlined values are calculated according to the predicted position of the NotI site.

Whole Genome mapping further confirmed the chromosome length polymorphism previously described for chromosomes I and II of the *B. microti* genome using PFGE analyses [5], although the difference between chromosome I of the R1 isolate and that of the Gray strain was found to be larger than that initially estimated by PFGE analysis (Fig. 4). The availability of the Whole Genome Maps for the R1 and Gray strains made it possible to position the NotI sites on those maps in relation with the KpnI sites. Using this analysis, the NotI restriction fragments carrying the chromosomal extremities were found to be in general agreement with the hybridization data using the telomeric probe (Fig. 1 and 5). In addition, comparative analyses of the maps of the R1 and Gray strains revealed the presence of new length polymorphisms on chromosome III and IV, which could not be resolved and detected by Southern blot analysis (Fig. 4). These polymorphisms include a 27 kb size difference on IVb, which is below the resolution of the PFGE analysis and therefore difficult to see on hybridization data (Fig. 1). Additional polymorphisms were found on IIIa, IIIb and IVa extremities, suggesting that major rearrangements take place at the end of the chromosomes in *B. microti*.

### Whole Genome Mapping revealed limited diversity in the core regions of the chromosomes

The availability of the WGM of the R1 and Gray strains, which were isolated from France and the United States (Nantucket, MA), respectively, led us to investigate possible differences between their genomes. Alignment of the Whole Genome maps of chromosomes I, II, III and IV from both strains showed no significant differences in the core region (Fig. 5) with most differences being within the detection limit of the Whole Genome Mapping method (5.3 kb for chromosome I, 3.7 kb for chromosome II, 0.6 kb for chromosome III and 5.9 kb for chromosome IV). These observations suggest that the DNA sequence composition associated with the core of the chromosome is very well conserved between the R1 and Gray strains.

Unlike the core regions of the chromosomes, major differences were found in the telomeric regions with size differences as large as 33.8 kb seen in the 5′ end of chromosome IV between the Gray strain and the R1 strain (Fig. 4). Notably, while this region of chromosome IV is shorter in the R1 strain compared to the Gray strain, the IIIa end of the R1 chromosome is larger than that of the Gray strain by an almost identical length (∼30.5 kb) (Fig. 4). Similarly, IVb in the R1 strain is ∼27 kb larger than that in the Gray strain, whereas the IIIb end of the R1 strain is ∼32 kb shorter than that of the Gray strain (Fig. 4). A similar length difference within and between chromosome telomeres and telomere-associated regions has been observed within and between *Plasmodium falciparum* isolates. [21]–[24]. The chromosomal ends play an essential role in maintaining antigenic diversity in parasites [25], [26]. Frequent duplication and recombination events in the telomere-associated regions result in gene families, many of which encode variable surface proteins, thereby generating antigenic variation [27], [28]. The completion of the genome sequence of *B. microti* led to the identification of a small set of gene families, including *B.microti* sero-reactive antigen family (*bmn*), and the *Tpr-like* and *vesa-like* families, at the chromosome ends [5]. It remains unknown whether the differences between strains in the 5′ and 3′ ends of the four chromosomes reflect recombination events taking place between chromosomes or are the result of independent expansions or contractions of telomeric repeats in each chromosome or a combination of these two events.

### Molecular evidence for a linear mitochondrial genome of *B. microti*

The first assembly of the mitochondrial genome of the *B. microti* R1 isolate indicated a circular 11.1 kb molecule containing two identical repetitive elements (inverted repeats: IR) [5]. Recombination events between these repetitive elements were proposed to result in two structural variants, the normal and inverted types [5]. More recently, Hikosaka and colleagues suggested that the mitochondrial genomes of the *B. microti* Munich strain and *B. rhodaini* are linear and exist in 4 different structural types resulting from recombination between two IR sequences, IR-A and IR-B [29]. In this configuration, only parts of the fragment encompassing the inverted repeats are present at both ends of the molecule. To re-examine the structure of the mitochondrial genome of the R1 and Gray strains, we performed detailed PCR and hybridization analyses using DNA isolated from red blood cells infected with these parasites and reassembled the existing reads from the genome project.

**Figure 6:**
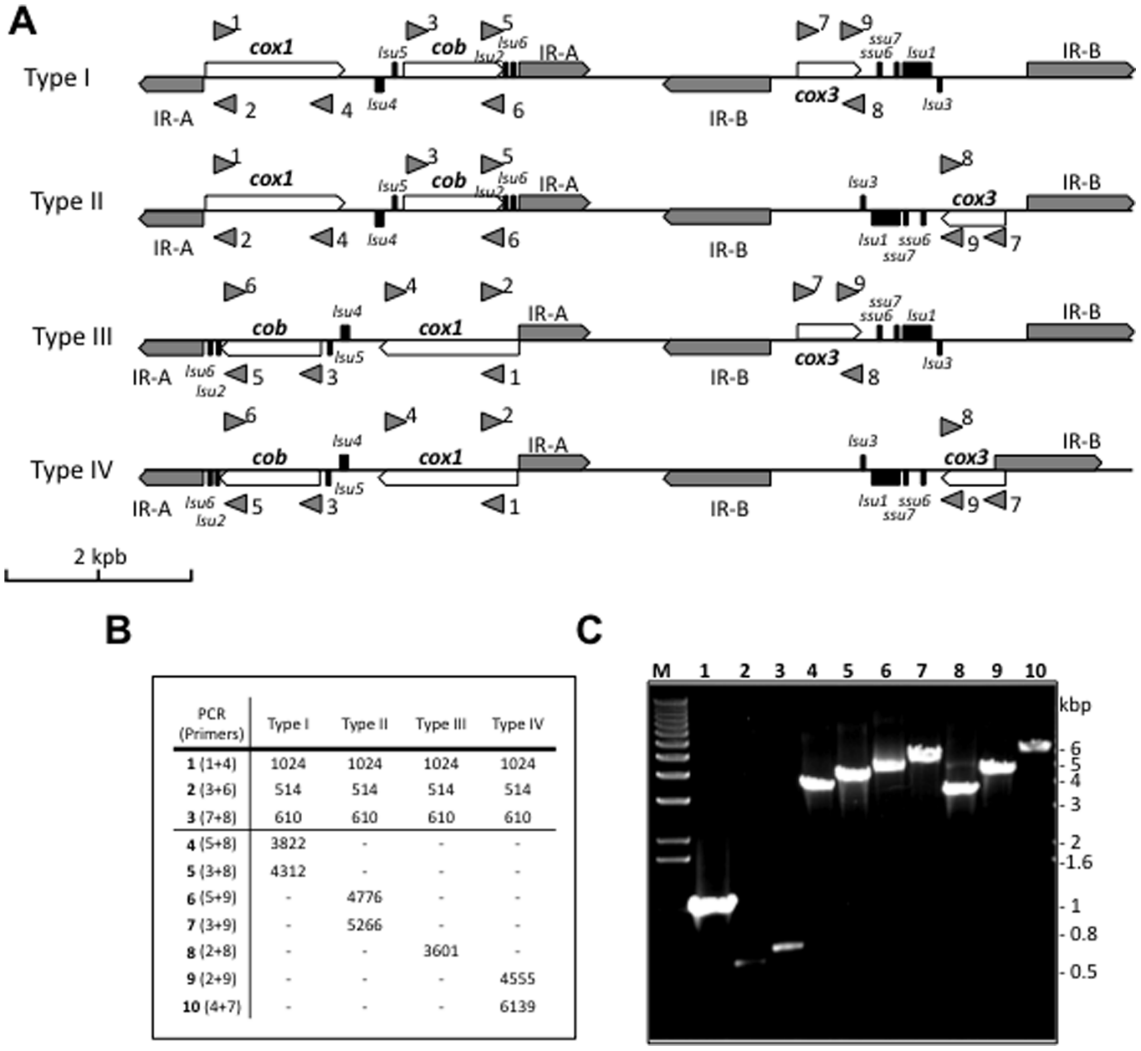
Evidence for a linear configuration and 4 structural types of the mitochondrial genome in *B. microti* by PCR analysis. **A.** Schematic representation of the linear configuration of the mitochondrial genome of *B. microti* and the 4 structural types. The mitochondrial chromosome carries three protein encoding genes: *cob* (cytochrome b), *cox1* (cytochrome c oxidase sub-unit 1) and *cox3* (cytochrome oxidase sub-unit 3). The ribosomal RNA genes are fragmented in apicomplexa (43, 44). Two genes encoding rRNA of the small ribosomal (*ssu6* and *ssu7*) and six genes encoding rRNA of the large ribosomal sub-unit have been characterized in *B. microti* (*lsu1, lsu2, lsu3, lsu4, lsu5* and *lsu6*). IR-A and IR-B represent the two independent inverted repeats. Arrows represent primers 1 to 9 (Table 1) used in PCR reactions to demonstrate the linear configuration and the 4 structural types of the mitochondrial genome of *B. microti.* **B.** Fragment sizes expected from PCR analyses for the 4 linear types I, II, III and IV. **C.** Agarose gel analysis of the different PCR products. The PCR fragments amplified are in agreement with the prediction.

To assess the linear structure and the 4-type model of the mitochondrial genome of *B. microti*, PCR analyses were performed using specific primers. Sense and their antisense PCR primers were designed to amplify the *cox1*, *cob* and *cox3* genes as well as the regions between them in both the forward and reverse strands (Fig. 6A). As expected, PCR primer pairs 1+4, 3+6 and 7+8 amplified *cox1*, *cob* and *cox3* genes, respectively, in both strains. In addition, combinations 5+8 and 3+8 demonstrated a Type I form of the mitochondrial genome; combinations 5+9 and 3+9 demonstrated a Type II form; combination 2+8 demonstrated a Type III configuration; and combinations 2+9 and 4+7 demonstrated a Type IV configuration (Fig. 6B and C). Interestingly each PCR reaction resulted in a single product corresponding to the predicted fragment of a linear mitochondrial DNA configuration. No additional fragments, 900 bp shorter in size (region between IR-A and IR-B) expected from a circular model could be detected (Fig. 6C). Together these PCR results suggest that the mitochondrial genome of the R1 strain is linear and exists in 4 structural types.

**Figure 7:**
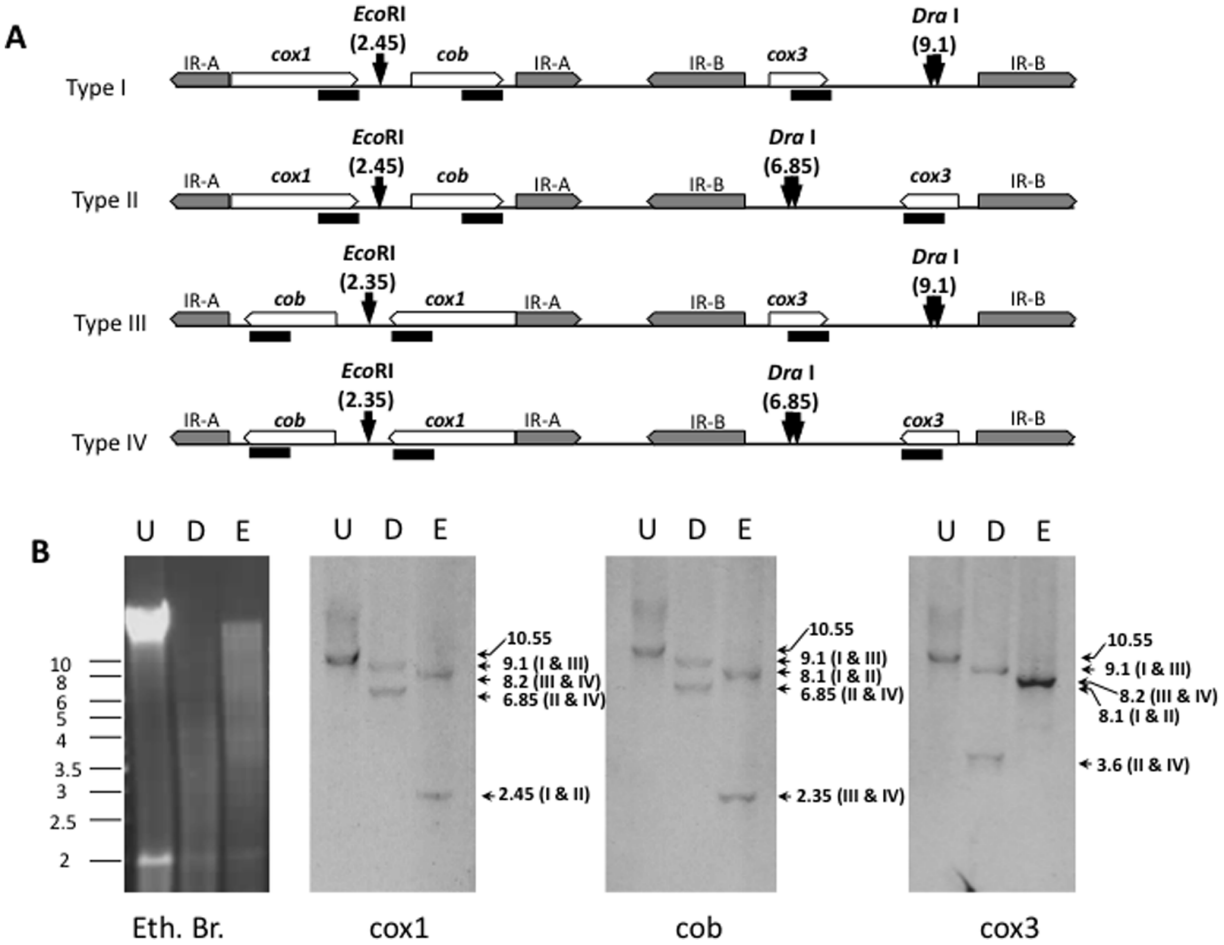
Evidence for a linear configuration and 4 structural types of the mitochondrial genome in *B. microti* by Southern analysis. **A**. Schematic representation of the linear configuration and the 4 structural types of the mitochondrial genome of *B. microti.* The protein encoding genes were used as genetic markers and target with specific probes (dark lines). Gene names are as described in Figure 6. IR-A and IR-B represent the two independent inverted repeats. DraI and EcoRI sites used to digest the genomic DNA and their positions on the molecules are represented. **B**. Southern blot analysis preformed with cox1, cob or cox3 probes (black bars) on of the R1 strain's mitochondrial DNA either undigested (U) or digested with DraI (D) or EcoRI (E). The theoretical sizes and associated types of mtDNA molecules are represented. Eth. Br.: Ethium bromide-stained agarose gel.

To further validate this configuration of the mitochondrial genome, genomic DNA from the R1 strain was digested with DraI or EcoRI and hybridized to specific probes corresponding to the *cox1*, *cob* or *cox3* genes. As shown in figure 7, hybridization of DraI-digested DNA with the cox1 or cob probes identified two bands, one of 9040bp in Types I and III, and another of length 6857bp, in Types II and IV. Hybridization of DraI-digested DNA with the *cox3* probe identified two bands of 9040bp from Types I and III, and another of 3608bp in Types II and IV, respectively. Hybridization of EcoRI-digested DNA with the *cox1* probe identified the two expected bands of 2457bp (Types I and II) and 8178bp (Types III and IV), whereas hybridization of EcoRI-digested DNA with the *cob* probe identified the expected bands of 8090bp (Types I and II) and 2369bp (Types III and IV) (Fig. 7). Similarly, hybridization of EcoRI-digested DNA with the *cox3* probe identified two bands of 8090bp and 8178bp expected from Types I and II, and III and IV, respectively (Fig. 7). Equally important, none of the DraI or EcoRI digestions resulted in a 10547bp band expected for a circular model of the mitochondrial genome. Together these results suggest that the mitochondrial genome of the *B. microti* R1 and Gray strains is linear, with the three genes arranged in the following order and orientation: (cox1  = >) – (cob  = >) – (cox3  = >), in type I; (cox1  = >) – (cob  = >) – (cox3 < = ), in type II; (cob < = ) – (cox1 < = ) – (cox3  = >), in type III; and (cob < = ) – (cox1 < = ) – (cox3 < = ), in type IV.

**Figure 8:**
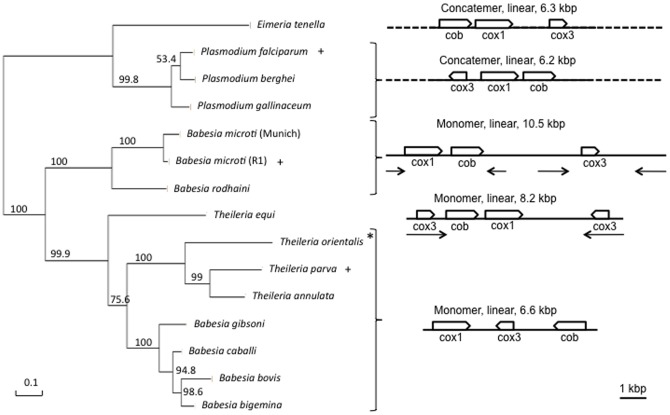
Phylogenetic analysis on the left panel is based on a combination of three protein sequences encoded by the genome of different apicomplexan mitochondria (cox1+cob+cox3). About 63% of the sites were conserved for the phylogenetic analysis. The scale indicates the inferred number of substitutions. Boostrap values are associated to each branch. The unrooted tree was calculated using the Phygeny.fr web site and default options of the one-click procedure. The schematic structures of mitochondrial genomes in apicomplexa (right panel) are based on species marked by a “+” in (A). Inverted repeats are represented by arrows. *T. parva* is presenting short inveted repeats that are not described in other *Theileria* and true-*Babesia* species. Dashed lines figure out the concatenated form of the molecule. * *T. orientalis* is presenting an inversion of the *cox3* gene. The gene order is highly conserved among *Plasmodium* species [47]. Variations are associated to rRNA genes and resolution site. The *P. falciparum* genome organization is also present in other species such as *P. floridensis*, *P. mexicanum* or *P. reichenovi*. A slight variation in the molecule structure of *P. berghei* and *P. gallineum* does not change the gene order (Fig. S3 in Supporting Information S1). This later organization is present in other species such as *P. fragile*, *P. knwolesi*, *P. sinium*, *P. vivax* or *P. yoelii*.

Consistent with the linear configuration of the mitochondrial genome of *B. microti*, *in silico* analyses revealed the presence of two inverted repeats, IR-A and IR-B, both in the middle and extremities of the mitochondrial genome. In the middle of the mitochondrial sequence, the inverted repeats are separated by a unique sequence of ∼900 bp (Fig. 6A). The IR-A and IR-B share no homology with each other but are similar to those described in the mitochondrial genome of the *B. microti* Munich isolate (Fig. S2 and appendices 1 & 2 in Supporting Information S1). These inverted repeats seem to be species-specific as no homology could be found between those of *B. microti* R1 and those of *Babesia rodhaini*, which is the closest species with a sequenced mitochondrial genome. The presence of four structural types is unique among apicomplexa and uncommon among known mitochondrial genomes [30], [31]. This feature further supports the results of previous phylogenetic analyses, which showed the *B. microti*-group (including *B. microti* Munich and R1 and *B. rodhaini*) to have diverged from the remaining piroplasms early in the evolution of the clade [6], [32]. Phylogenetic analyses based on mitochondrial genes provided further evidence that *B. microti* defines a new lineage in the apicomplexan phylum (Fig. 8).

## Discussion

**Figure 9:**
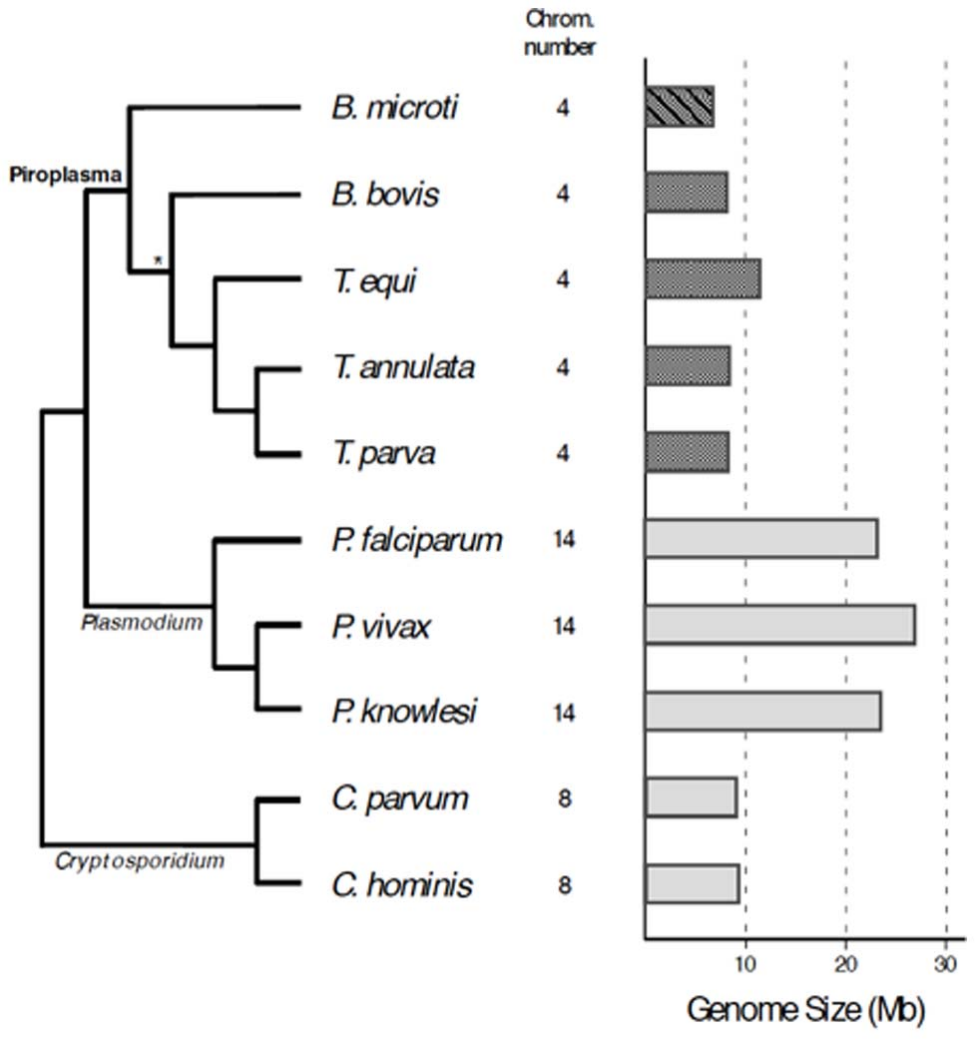
Genome size and chromosome number in apicomplexan species. Number of nuclear chromosomes and genome size are shown in panel on the right. Piroplasma taxa shown in dark color; *B. microti* stripped. Schematic of phylogenetic relationships among species shown on the left. Resolution of the interior node in the piroplasma clade marked with ‘*’ resolved according to [34], based on study with 150 nuclear genes. Resolution differs from that obtained with mitochondrial sequences (Fig. 8) and the 18S rDNA gene [6].

Whole genome mapping and hybridization data described in this report provide strong evidence that the karyotype of *B. microti* is composed of four chromosomes. This configuration is consistent with the presence of four centromeric-like regions at median or sub-median positions on chromosome II, III and IV and the distal position on chromosome I [5]. The presence of four chromosomes is a conserved feature among most piroplasms (Fig. 9, [33]). Recent sequencing efforts have shown that the genomes of *Theileria equi* [34] and *T. orientalis* [35] contain 4 chromosomes. These two organisms are important links in the genomic evolution of the Theileridiae family. Their respective sizes (11.6 and 9 Mb) are considerably larger than those of *T. parva* and *T. annulata* (8.3 Mb) [36], [37]. *Babesia bovis* is the only member of the true-*Babesia* clade for which a genome sequence has been reported [38]. Its 8.2 Mb genome also consists of four chromosomes. The only exception among piroplasms for the 4-chromosome genome configuration appears to be *B. canis* [15], which belongs to the so-called “large *Babesia”* and has five chromosomes and a relatively large genome size of 15 Mb. *B. microti* and *B. bovis* belong to the so-called “small *Babesia”* group. However, this group is paraphyletic and all phylogenetic analyses performed so far support distinct lineages for these two species and highlight the need to rename *Babesia microti*.

Whole Genome Mapping further showed that the core regions of the *B. microti* chromosomes are highly conserved between the R1 and Gray strains characterized in this study. In contrast, the telomeric and subtelomeric regions of the chromosomes of these two strains vary considerably in length. The structure of the subtelomeric regions has been partially sequenced and consists of a mosaic of repeats found in variable copy numbers and locations (Fig. S5 in Supporting Information S1). Some of these mosaic elements encode known and putative surface antigens [39], [40]. Whether or not these gene families differentially distribute in the telomeric and subtelomeric regions of the R1 and Gray chromosomes awaits better sequencing coverage of these regions. Such information is of particular interest as it might indicate differences in pathogenicity between strains and may serve as genetic markers to map the diversity of *B. microti* isolates and their geographic distribution.

Dynamic chromosome ends are common features in all eukaryotes. Several processes protect the genome from telomere loss and deleterious rearrangements due to ectopic recombination at chromosome ends. Most of these genetic events result from double strand breaks (DSB) taking place frequently in the subtelomeric regions. These breaks are subsequently repaired by either Non-homologous End Joining (NHEJ) or Homologous Recombination (HR) associated pathways [41]–[43]. Analysis of the annotated genome of *B. microti* showed that candidate genes encoding NHEJ enzymes are not present in this parasite [5]. This suggests that the Break Induced Replication (BIR) pathway, which is associated with homologous recombination and known to play an essential role in telomere recovery and mosaic organization of chromosome ends [25], [41], [43], [44], may be responsible for most of the polymorphism observed between the R1 and Gray isolates. Our analysis of WGM data showed, however, two exceptions to this genetic model. The first is manifested by a rearrangement associated with the IIIa end (Fig. S6 in Supporting Information S1). This rearrangement covers a large fragment that includes 50 kb of the 5′ region of the FO082874 supercontig (Fig. 4). Several unique genes are found in this region and encode proteins such as the SEC24 subunit of the COPII coat (BBM_III00030), the cytochrome c oxidase 2a subunit (BBM_III00045), the RNA pol II RPB11 subunit (BBM_III00075) and the ribosomal protein L27a (BBM_III00105), predicted to play an essential role during the parasite intraerythrocytic life cycle. In the case of this rearrangement, the differences between the WGM of the Gray and R1 strains may be due to large reshuffling of the chromosomal ends or possibly from a translocation event. The second event is in the form of a duplication of a region between IIIb and IVa (Fig. 3). This region is in one orientation on chromosome III and on the opposite orientation on chromosome IV (region S9 in Fig. S5 in Supporting Information S1). The duplicated region only partially encompasses the IIIc region containing repeated blocks of chromosome end specific sequences (including a member of the *bmn* multigene family, BBM_III04855) but also includes a gene encoding a putative asparagine synthetase (BBM_III04860) and a hypothetical gene (BBM_III04865). The breakpoint is proximal to a gene encoding a putative aconitate hydratase 1 (BBM_III04870).

We have performed phylogenetic analyses based on the three proteins encoded by the mitochondrial genome (Fig. 8). These analyses confirmed the previous placement of *B. microti* in a separate lineage among apicomplexa [5]. Our PCR and Southern blot analyses further demonstrated that the mitochondrial genome of *B. microti* is linear and exists in four different types (I to IV) (Fig. 6 and 7 and Fig. S1 in Supporting Information S1). Therefore, *B. microti* presents an organization distinct from any of the known mitochondrial genome structures described so far in piroplasmida (Fig. 9B). The gene arrangement in the linear mitochondrial genomes of *Babesia sp*. other than *B. microti*, namely *B. gibsoni, B. bigemina* and *B. caballi,* is found to be highly conserved: (*cox1*  = >) – (*cox3* < = ) – (*cob* < = ) [26]. The same gene arrangement has been found in *T. parva* and *T. annulata* (Fig. 8), although in *T. orientalis* the cox3 is in reverse orientation [45]. Differences in gene order and orientation provide insights into the evolution of piroplasmida, and suggest that significant rearrangements took place in the mitochondrial genomes since these species diverged from their common ancestor (Fig. S3 in Supporting Information S1). Changes in gene order between *B. microti* and *T. equi* require at minimum one inversion of *cob* and *cox1* gene position ([(cox1 = >) – (cob = >)] and [(cob = > – (cox1 = >)]) respectively). Remarkably, the relative order of *cob* and *cox1* genes is not modified in the four structural types of the *B. microti* mitochondrial genomes (Fig. 5 and 6). Assuming that the gene organization in *T. equi* represents that present in its common ancestor with the lineage leading to other *Theileria* and the true-Babesia species, then a large inversion encompassing the *cox1* and *cox3* genes took place in the latter lineage, combined with a size reduction of the mitochondrial genome that correlates with the loss of inverted repeats.

Inverted repeats may play a major role in the evolution of piroplasmida mitochondrial genomes and could help fulfill functions such as replication and segregation of the linear molecule. The mitochondrial genome of piroplasmida is characterized by a monomeric structure whereas it is multimeric and organized in a concatemer in *Plasmodium* and *Eimeria* species (Fig. 8, [45]–[50]. The concatemer structure is produced by replication that subsequently leads to a single molecule following recombination [45], [47], [49]. Large inverted repeats are present in *B. microti* and *T. equi* but are lacking in most other piroplasmida with the exception of *T. parva* where the repeated sequences are only 60 bp long and scattered at chromosome extremities [47].

Characterization of genes involved in the maintenance of the mitochondrial genomes of apicomplexa may help better define the importance of inverted repeats in the evolution of these genomes. We hypothesize that the evolution of mitochondrial genomes of piroplasmida may have occurred in several steps based on phylogenetic data (Fig. 8). First, inverted repeats may have originated from recombination events between two concatenated copies of the mitochondrial genome (Fig. S4 in Supporting Information S1) and distributed in both the middle and ends of the linear mitochondrial genomes. Second, repeats in the middle of the molecule are lost (Fig. S3 in Supporting Information S1). Third, repeats at chromosome ends degenerate. Further studies are needed to understand the mechanism of replication of the mitochondrial genome of *B. microti*, the functional and evolutionary significance, if any, of the unusual configuration of this genome and the link between the DNA repair machinery of *B. microti* and the recombination of mtDNA into 4-types.

In conclusion, the Whole Genome mapping and hybridization analyses of the *B. microti* genome presented here provide conclusive evidence for the presence of four nuclear chromosomes and highlighted significant length differences between *B. microti* strains in the telomeric and sub-telomeric regions but high conservation in the core regions of the chromosomes. Our molecular analyses further demonstrates that *B. microti* contains a linear mitochondrial genome with four structural types. The unique genomic structure, evolution and metabolic properties of *B. microti* revealed by genome sequencing, mapping and annotation will set the stage for the development of better strategies for treatment of human babesiosis.

## Supporting Information

Supporting Information S1 (PDF)
