# Supplementary material for: Whole Genome Mapping and Re-Organization of the Nuclear and Mitochondrial Genomes of Babesia microti Isolates
Source: PLoS One. 2013 Sep 4;8(9):e72657. doi: 10.1371/journal.pone.0072657 (PMC3762879; doi:10.1371/journal.pone.0072657)
Supplement: Supporting Information S1 — (PDF) [file pone.0072657.s001.pdf]

Figure S1

Type I

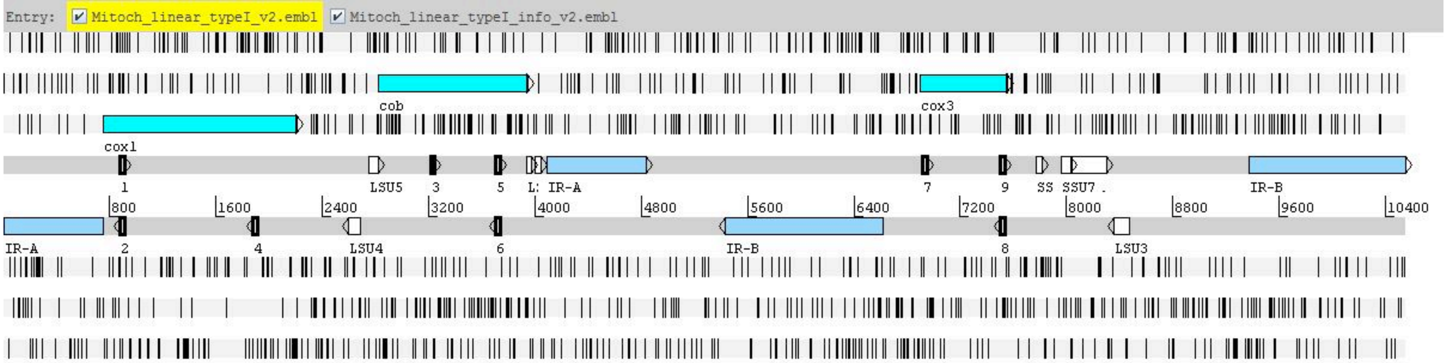

Type II

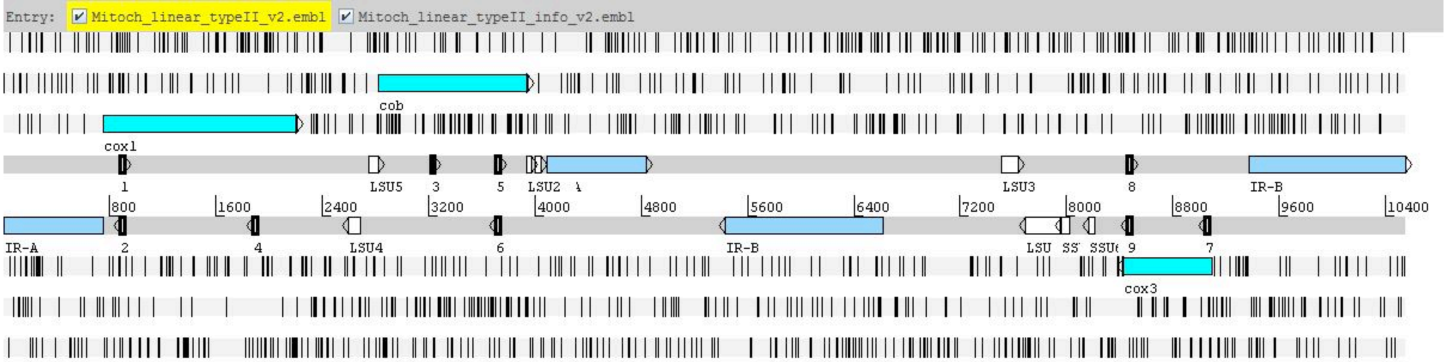

Type III

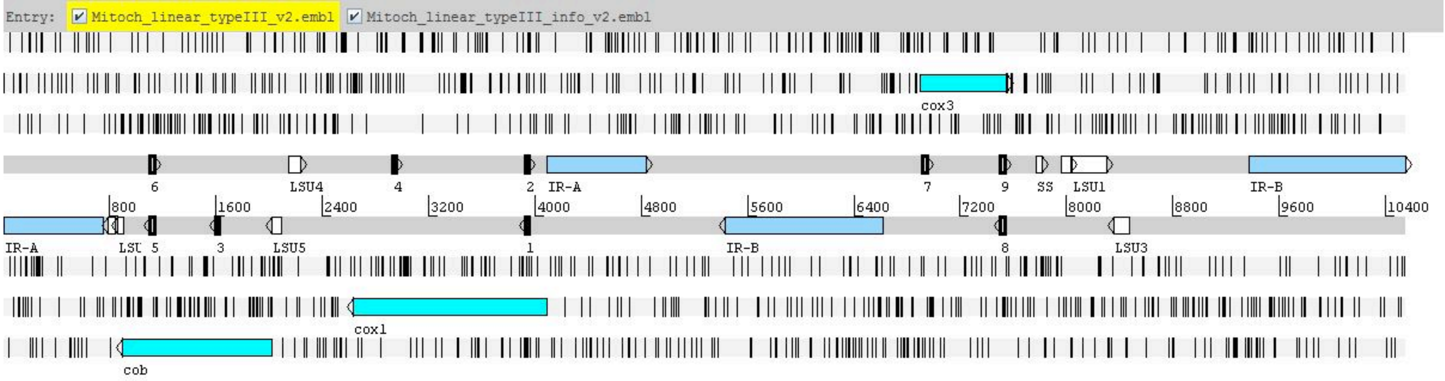

Type IV

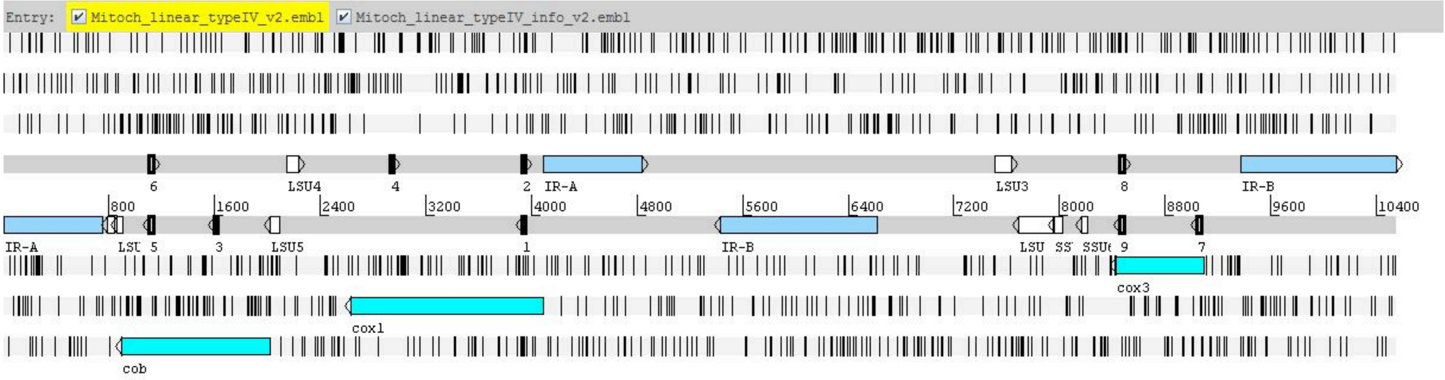

Figure S2

A.

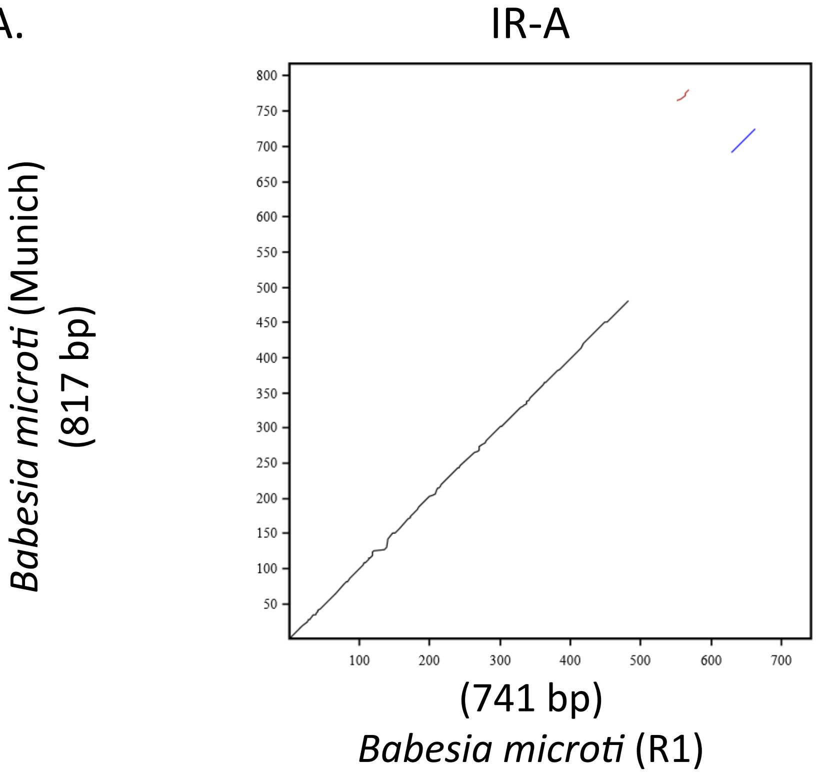
IR-A
Babesia microti (Munich)
(817 bp)
800
750
700
650
600
550
500
450
400
350
300
250
200
150
100
50
100
200
300
400
500
600
700
(741 bp)
Babesia microti (R1)

B.

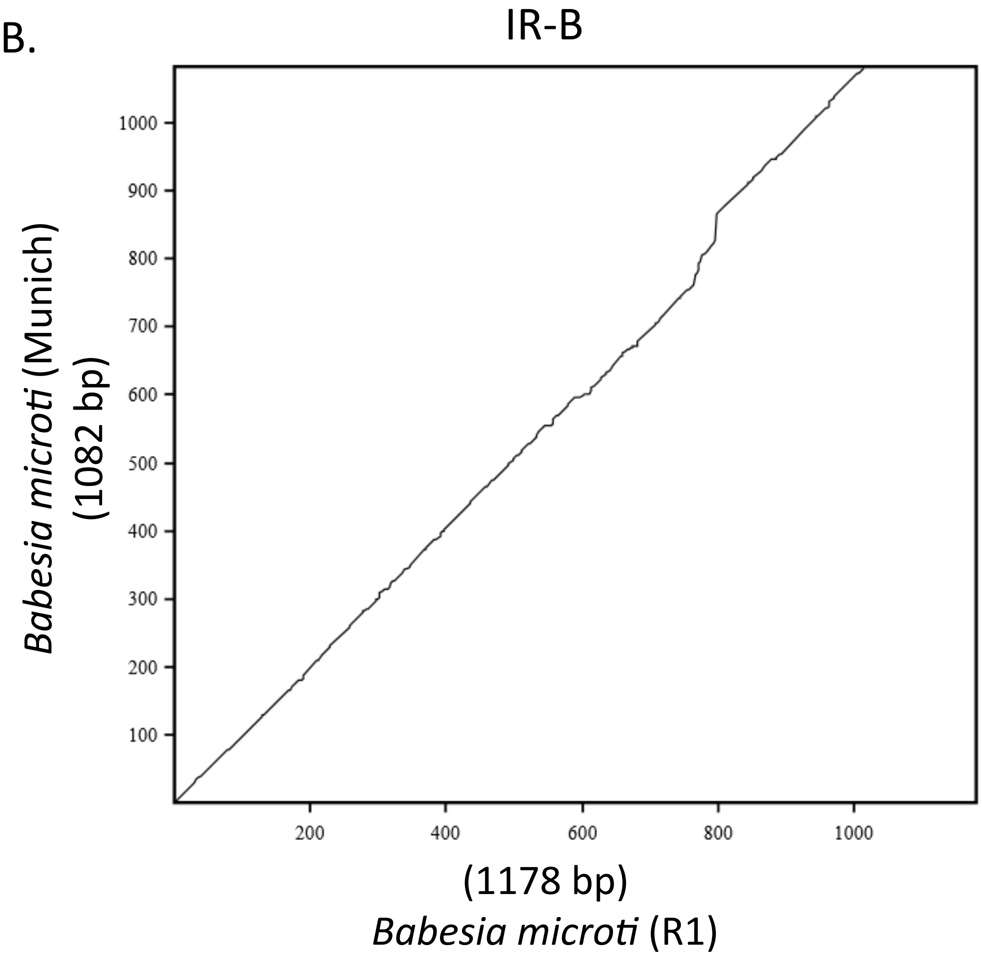
IR-B
Babesia microti (Munich)
(1082 bp)
1000
900
800
700
600
500
400
300
200
100
200
400
600
800
1000
(1178 bp)
Babesia microti (R1)

Figure S3

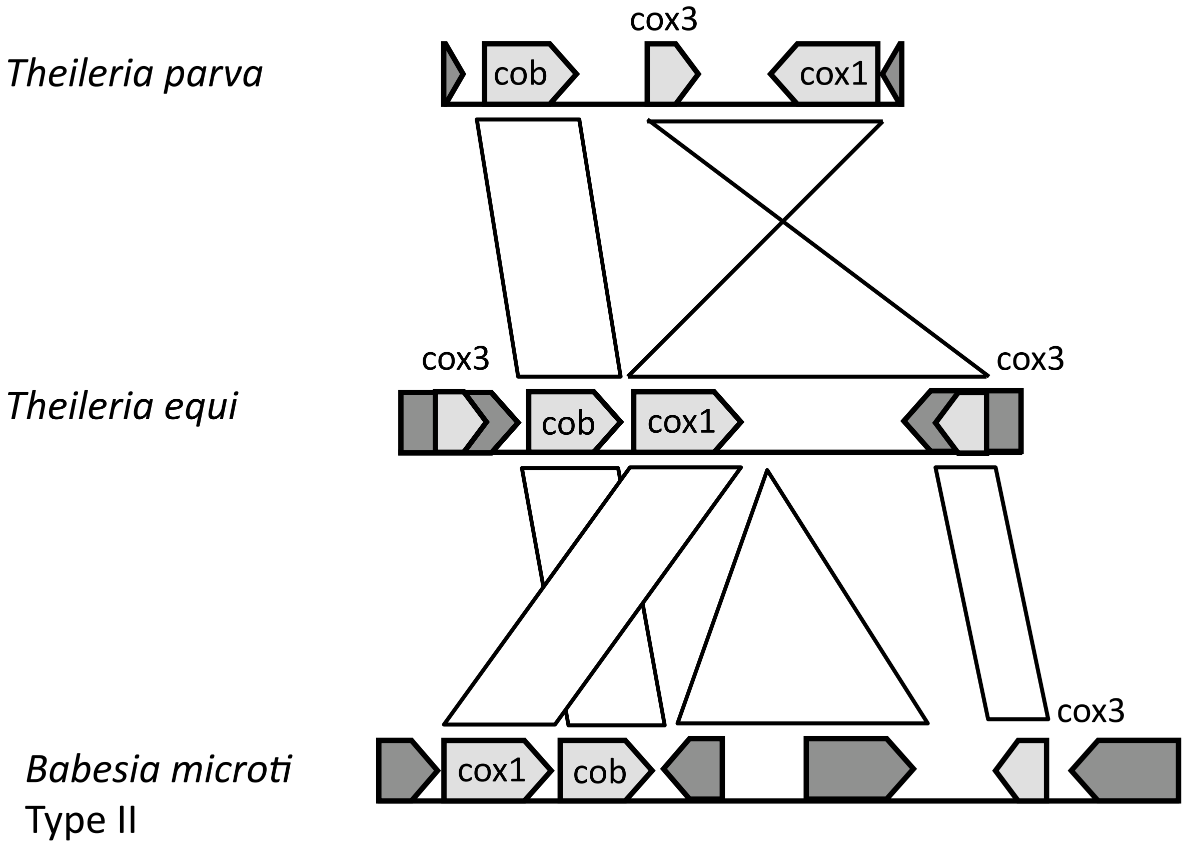

Theileria parva
cox3
cob
cox1
Theileria equi
cox3
cox3
cob
cox1
Babesia microti
Type II
cox1
cob
cox3

Figure S4

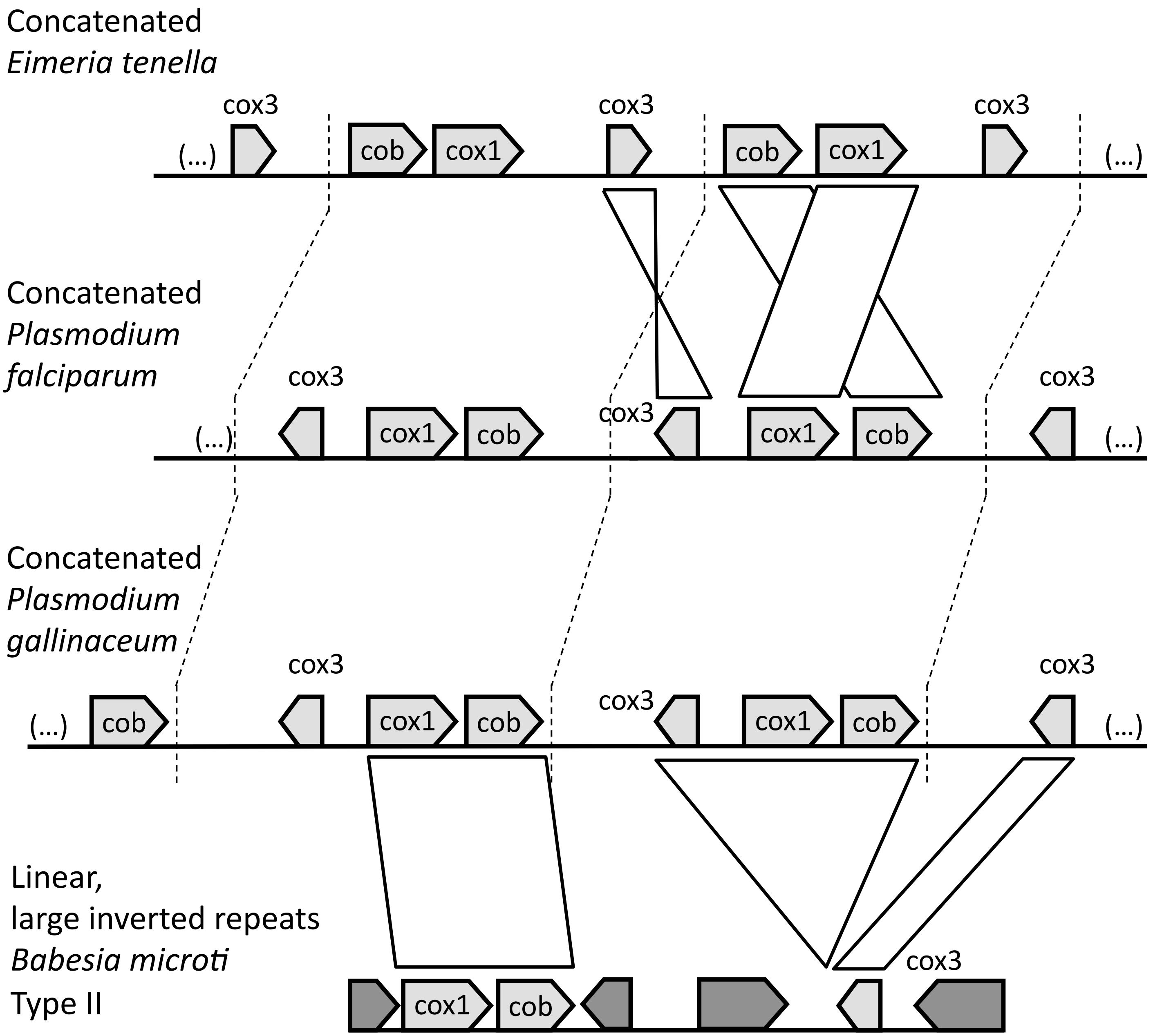
Concatenated
Eimeria tenella
cox3
cob
cox1
(...)
Concatenated
Plasmodium falciparum
Concatenated
Plasmodium gallinaceum
Linear,
large inverted repeats
Babesia microti
Type II

Figure S5

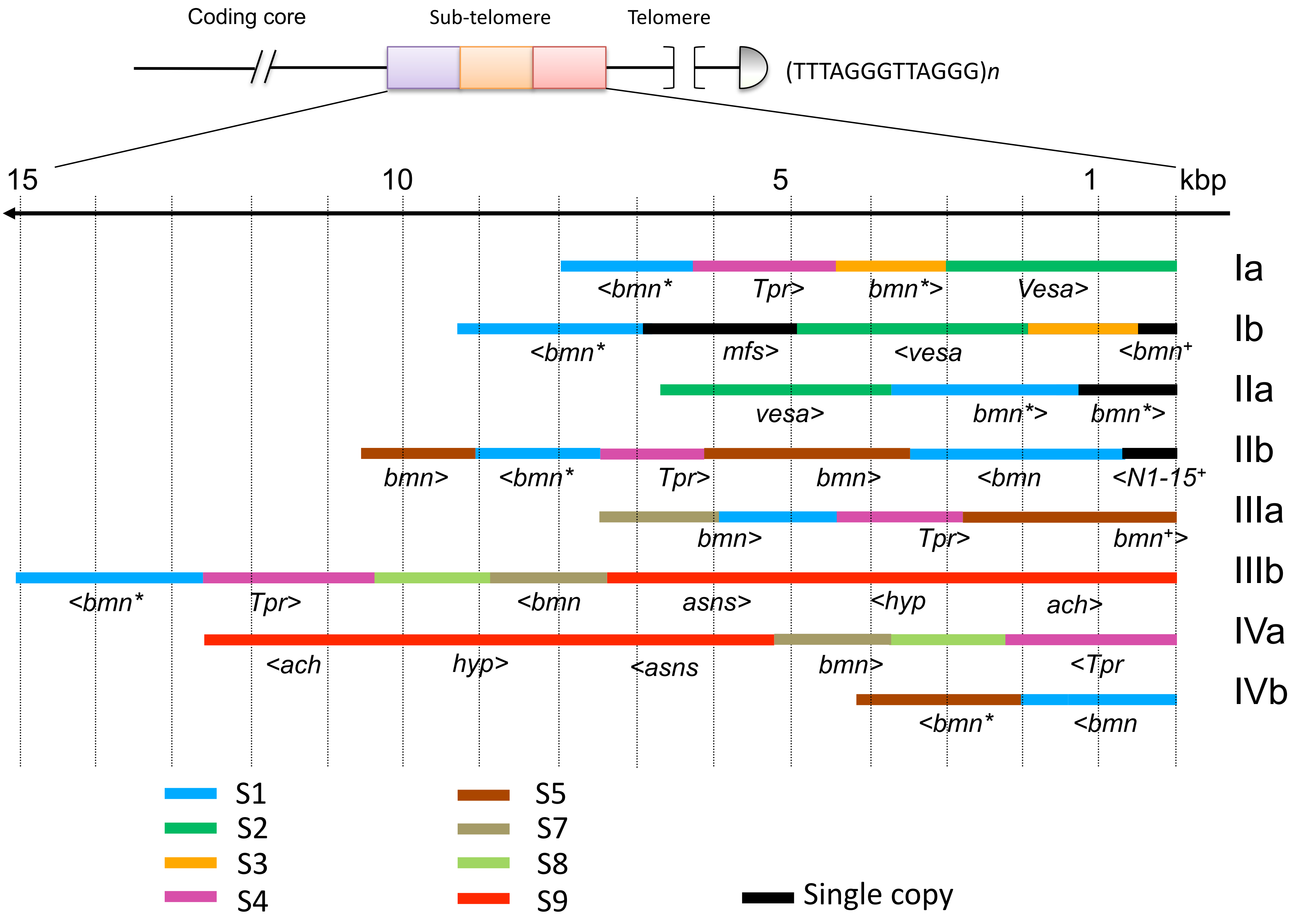
Coding core
Sub-telomere
Telomere
(TTTAGGGTTAGGG)n
15
10
5
1
kbp
Ia
<bmn*
Tpr>
bmn*>
Vesa>
Ib
<bmn*
mfs>
<vesa
<bmn+
IIa
vesa>
bmn*>
bmn*>
IIb
bmn>
<bmn*
Tpr>
bmn>
<bmn
<N1-15+
IIIa
bmn>
Tpr>
bmn+>
IIIb
<bmn*
Tpr>
<bmn
asns>
<hyp
ach>
IVa
<ach
hyp>
<asns
bmn>
<Tpr
IVb
<bmn*
<bmn
S1
S2
S3
S4
S5
S7
S8
S9
Single copy

Figure S6

Comparative Genomics of *B. microti* strains IIIa region
Chr III- 5’ region of the R1 and Gray isolates
Whole Genome Map performed with *Kpn*I enzyme.

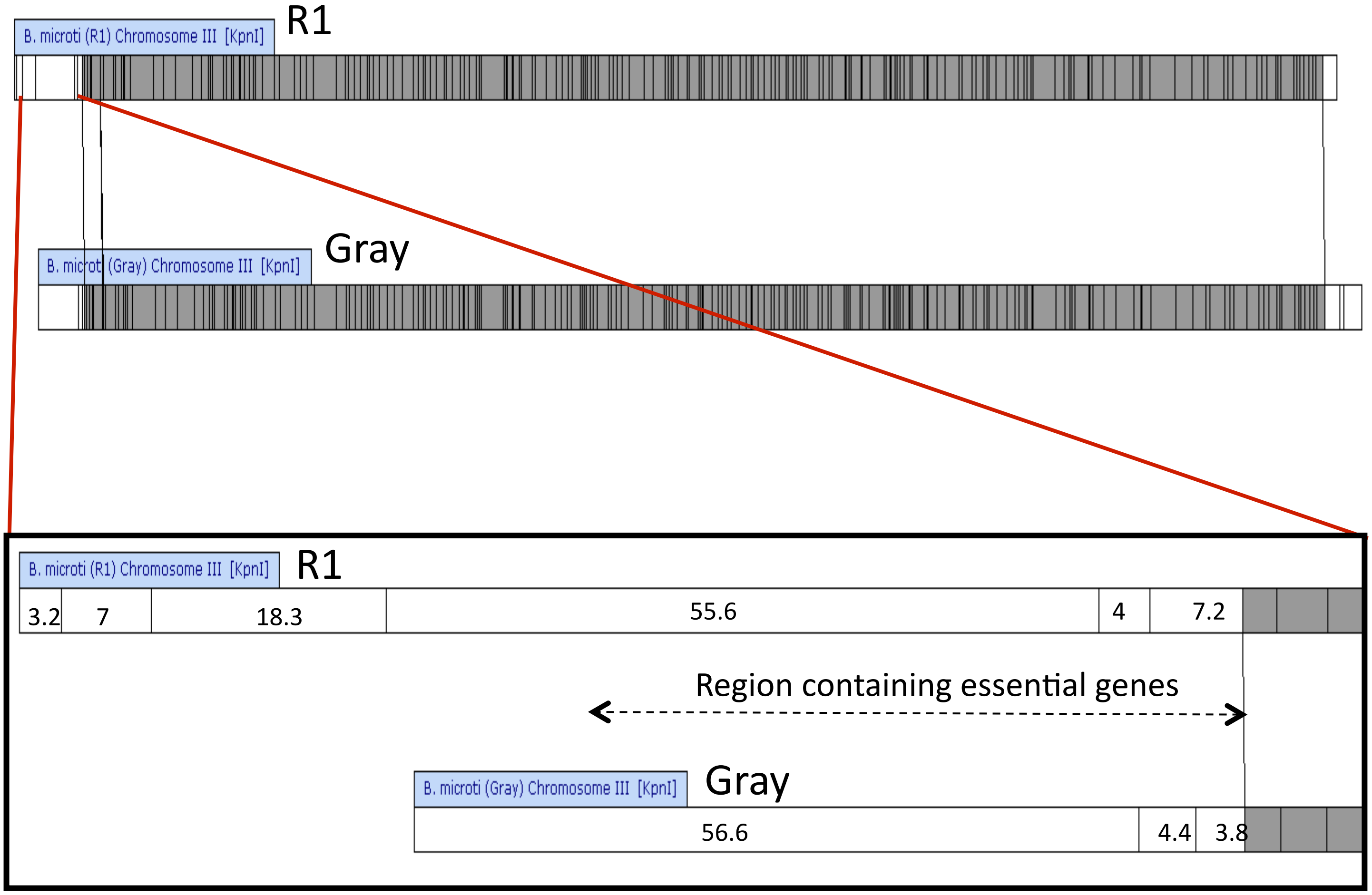

Figure S1. *B. microti* mitochondrial genome annotation and distribution of the genetic information among the four different types. The graphical output was obtained using Artemis (Sanger).

Figure S2. FASTA comparison of the IR-A and IR-B inverted repeats mapped to the mitochondrial genomes of *B. microti* R1 and Munich isolates. Dot plots represent the best local alignments. They were generated using Lalign software: http://fasta.bioch.virginia.edu/fasta_www2/fasta_www.cgi?rm=lalign
See appendix 1 for IR-A alignment and appendix 2 for IR-B alignment.

Figure S3. Comparison of the *B. microti* mitochondrial genome organization with that of *T. equi* and *T. parva*. Light grey boxes represent the protein coding genes. Dark grey represent inverted repeats.

Figure S4. Comparison of the linear monomeric *B. microti* mitochondrial genome organization with the concatenated mitochondrial genomes of *E. tenella*, *P. falciparum* and *P. gallinaceum*. Light grey boxes represent protein coding genes. Dark grey boxes represent inverted repeats.

Figure S5. Mosaic organization of *B. microti* subtelomeric regions based on the partially sequenced chromosome ends of the R1 strain [1]. S1 to S9 represent subtelomeric regions that share significant homology with each other. These regions contain putative protein coding genes including *Bmn*, *Tpr*-like (S4) and *vesa*-like genes (S2). Orientation of genes is provided by the < and > signs. Putative pseudogenes are indicated by *. Incomplete genes at chromosome ends are marked by +. Full length copies of *bmn* genes are also found in the coding core of the chromosomes. The IIIb and Iva sub-telomeric regions contain the same set of putative essential genes in opposite orientation (S9). These genes are: *asns*, asparagine synthetase; *ach*, aconitase hydratase; *hyp*, hypothetical protein. The *N1-15* gene is highly homologous to a gene encoding the N1-15 surface antigen characterized in the MN1 *B. microti* isolate [2]. *Mfs*: transporter of the major facilitator superfamily.

Figure S6. *Kpn*I optical map of the 5-prime region of chromosome III contig resulting from Whole Genome Mapping of *B. microti* R1 and Gray strains (IIIa extremity). The difference between the two maps includes a ~50 kbp region on the contig KIII_ctg350 (database entry

FO082874) from the first release of *B. microti* R1 genome sequence. This region encompasses several essential genes.

References.

1. Cornillot, E., et al., *Sequencing of the smallest Apicomplexan genome from the human pathogen Babesia microti.* Nucleic Acids Res, 2012. **40**(18): p. 9102-14.
2. Lodes M.J., et al, Serological expression cloning of novel immunoreactive antigens of *Babesia microti*. Infect Immun. 2000 May;68(5):2783-90.

Table S1. Size differences according to Whole Genome Map of *B. microti* R1 and Gray isolates.

	R1	Chr.	Gray	
	1 378.451	I	1 346.113	
204.891				252.909
	1 583.342	II	1 599.022	
269.046				254.668
	1 852.388	III	1 853.690	
23.169				22.825
	1 875.557	IV	1 876.515	

Gray - R1
-32.338
15.680
1.302
0.958

Appendix 1.

Best local alignment between the R1 and Munich IR-A repeats. It was performed with the blastn2 matrix. The alignment was performed with LAlign software at web site: http://fasta.bioch.virginia.edu/fasta_www2/fasta_www.cgi?rm=lalign

```
Waterman-Eggert score: 414;  167.9 bits; E(1) <  1.7e-45
73.9% identity (73.9% similar) in 532 nt overlap (2-481:1-480)

R1     GCATTCGCTCATA-CGTACATAATA-TCTTATGGTCCT--ATA-GTACACCACAAGCATA
       ::::::::::::: : : ::::::: : :::::::  :  ::: : ::::::::: : ::
Munich GCATTCGCTCATAACCT-CATAATAGT-TTATGGT--TTAATAAG-ACACCACAAACCTA
               10         20         30          40         50

         60         70         80         90       100        110
R1     TTATCTAC-TATAAAGTGATGTA-CATACACT-CACTGCACTACAATATACC-TACATGT
       :::::: : :::::::::::::: :: ::::: ::::::::::::::::::: : :::::
Munich TTATCT-CGTATAAAGTGATGTAACA-ACACTTCACTGCACTACAATATACCAT-CATGT
          60         70        80         90       100        110

                  120       130                  140       150
R1     -TAATC-----CATGTTTTGTTTTGGGTTAAA-----------GAAACATGTATGGTAAT
        :: ::     :::              ::::           :::::::::  ::::::
Munich ATA-TCTCCTTCAT--------------TAAAAGGGGGTAGTCGAAACATGT--GGTAAT
             120                     130       140         150

          160       170         180        190       200
R1     -TGATAGTATACATATC-ACAATACT-AGC-ATCGTGCTGGATTGAGAACCAATTA----
        : :::::: :: :: : :::::::: : : :: :: ::  :: ::::    ::::
Munich GTTATAGTAAACCTA-CTACAATACTTAACTATTGTACTACATAGAGA----ATTATTTG
         160       170        180       190       200           210

       210        220       230       240        250       260
R1     ACGGAGG-AAGTTTATGCTTCTTCAATAGGTACGA-TAGGCCTCGTTTTGTATACTGTTA
       ::: : ::::  :::::::::: :::::::: : ::::  :::::::::::::::
Munich ACG-ATGTAAGTCAATGCTTCTTCTATAGGTAC-ACTAGGTATCGTTTTGTATACTG---
               220       230       240        250       260

             270          280        290       300       310
R1     CGGT----CTACAAAGC---TTCAAGAG-TCTGTGAAAGCTTCTAGCTTGATTGACTATT
         ::    :::::   :   ::::: :: ::::::::::::: : :::::::::::::::
Munich --GTAAAACTACA---CCGTTTCAA-AGGTCTGTGAAAGCTT-T-GCTTGATTGACTATT
           270          280        290       300         310

       320       330         340        350       360        370
R1     TGCTAGCAGTTAAGACTAA--CAGA-TATGTCTAACGCAGATTGTGT-ACATACCAAAGC
       ::::: :::::: :  :::  :: : ::::: :::::::: :::::: : ::: ::::::
Munich TGCTAACAGTTA-G--TAAACCATAGTATGTTTAACGCAGCTTGTGTGA-ATAACAAAGC
       320        330         340       350       360        370

           380       390       400       410          420       430
R1     AGCTTTATATGGTATTCACTGGGATATGTGGTACTCAGACA--AAGA-TGACTCTCTACA
       : :::::: : ::::::::: ::::: ::::::::::::::  : :: : ::::::::::
Munich AACTTTAT-TAGTATTCACTAGGATACGTGGTACTCAGACACGAGGACT-ACTCTCTACA
           380        390       400       410       420        430
```

```
                 440       450       460       470       480
R1     CATAT-TCTGAGGGATAGTTAGTGCTACAGTTATAGACTAGCTAAAACAGTT
       : ::: : : :: :::: :  :::::::::: :::::: :::::: ::::::
Munich CGTATGTTTTAGTGATA-T--GTGCTACAGTAATAGACAAGCTAATACAGTT
               440          450       460       470       480
```

Appendix 2

Best local alignment between the R1 and Munich IR-B repeats. It was performed with the blastn2 matrix. The alignment was performed with LAlign software at web site: http://fasta.bioch.virginia.edu/fasta_www2/fasta_www.cgi?rm=lalign

```
Waterman-Eggert score: 972;  401.7 bits; E(1) <  1.5e-115
70.4% identity (70.4% similar) in 1191 nt overlap (1-1013:1-1079)


R1     ATAGATTAGTACTTGTACTAAGCTACATAA----TAGGCTATAGTAGCATATAGAATTAC
       :::::::::::::::::::::::::: :::    ::: :    :::::::::::::::::
Munich ATAGATTAGTACTTGTACTAAGCTAC-TAACCTTTAG-C----GTAGCATATAGAATTAC
               10        20         30             40        50

         60        70        80         90       100       110
R1     TTGTTAGTTCTTCTATAGGTACAAC-ACTATCTATTCGCAGTATATGTAGTGGTGTAGCT
       ::::::::::::::::::::::: : :::: ::::::::::::::::::::::::: :::
Munich TTGTTAGTTCTTCTATAGGTACA-CTACTAACTATTCGCAGTATATGTAGTGGTGTGGCT
           60        70         80        90       100       110

         120        130       140       150       160       170
R1     ATTGCAGATACAT-ACGCATGTAATAGATGTGCGGCAGTACTAAGGTGCCTACAACTAA-
       :::: :::::::: :: :::::: ::::: ::::: :::::::::::::::::  ::::
Munich ATTGTAGATACATCAC-CATGTATTAGATATGCGGTAGTACTAAGGTGCCTAC--CTAAC
           120        130       140       150       160         170

            180            190       200       210         220
R1     -TACCATAACGTATCAT-----TACGTTAATATATCCTAAAGT-ATCAATA-GATGCTTT
        :::::::::     ::     :: :::::::: ::::: ::: ::: ::: ::: :: :
Munich ATACCATAAC-----ATAGAGATATGTTAATATGTCCTAGAGTCATCGATAAGATACTCT
              180            190       200       210       220

          230       240       250        260        270        280
R1     ATG-ATAACTCCATTAGTACATAGGTATACTAG-TACATG-TATTGCTACTAGC-AGGTA
       ::: ::::::::::::::::::::::::::::: :::::: :: :::::::::: :: ::
Munich ATGGATAACTCCATTAGTACATAGGTATACTAGATACATGATAATGCTACTAGCTAG-TA
         230       240       250       260       270       280

             290        300               310          320
R1     ACTATATATAGTGAC-TAGCA------CTATGCTACTACTACC-----ATACCATACAAT
          ::: ::: :::: :: ::      ::::: :     ::::     :::   ::::::
Munich ---ATACATACTGACATA-CATGTCATCTATG-T-----TACCTCATTATA---TACAAT
             290        300       310             320          330

     330           340            350       360        370
R1     GATTAGA----TGGTCCGGTA-----GAGATCATTGTATCCCATGCTAT-GATATGTGTT
        :: :::    :       ::     ::::: ::::::::::::::: : ::::::::::
Munich AATAAGACTTAT-------TAAACAAGAGATTATTGTATCCCATGCT-TAGATATGTGTT
             340              350       360       370        380

     380       390           400       410       420       430
R1     AGTTGATCCAA--TCAACTA--TTAATGCACAATATCAACAGATTATTGCGATTAACTA-
        ::  ::: ::  : :: ::  ::::: :::::: :: : :::: ::::::::::: ::
Munich TGT--ATCAAACATTAA-TACCTTAATACACAATGTCTATAGATGATTGCGATTAAGTAA
             390        400       410       420       430       440
```

```
          440       450        460       470       480       490
R1     CAAATCAACTCGCATGTAACGAT-ATGTCGTGATAACATCGTACAGATGTCACTAAT-AA
       ::::::::: ::::::::::::: ::::::: : :::::::::: :::::::::: : ::
Munich CAAATCAACCCGCATGTAACGATCATGTCGTAA-AACATCGTACTGATGTCACTA-TGAA
              450       460       470        480       490

             500       510        520       530             540
R1     GCGTA-TACCATGATACCC-AGAGGTACAAACTAGCAACTA--GT----TAGGGTTAAAG
       ::::: ::::: : ::::: :: :::::::  ::: :::::  ::    :::   :: ::
Munich GCGTAATACCA-G-TACCCCAGCGGTACAAT-TAGGAACTAATGTCTATTAG---TAGAG
      500        510        520        530       540          550

          550              560        570        580       590
R1     CTGCAAAGCTTG------A-TATCCTTGAC-TGCCCGCTGTG-TGAGCTGACGTGGCCTA
       :       ::::      : :::   :::: : :: :::::: : :: : ::::     :
Munich C-------CTTGTGCTTTACTAT---TGACCTACCAGCTGTGCT-AGGTAACGT-----A
                   560          570       580        590

         600       610           620          630        640
R1     TGCCGCTGTAGATATAC----AGATGGCAGGGAG-A--TAAGCCA-GTACCAACG---TA
           ::::     ::::    ::: :  ::: :: :  : ::::: :   ::: :   ::
Munich ----GCTG-----ATACCTCGAGAAG--AGGTAGCAGTTTAGCCATG---CAAAGCTATA
            600            610         620       630          640

          650          660       670       680           690
R1     AGAGCTTAGCTTA-T--CAGTCACGTGGTATAATGGTCG----CTAATAGTTAGCAGTTA
       :::::: : :::: :  :::: ::  ::: ::     ::    :: :: :::::::::::
Munich AGAGCTAAACTTAGTACCAGT-AC--GGT-TA-----CGGTTACTTATTGTTAGCAGTTA
            650       660           670            680       690

       700        710          720       730       740        750
R1     CCACTACTT-GTATAC--TGTAA-GTGTATAAGTGGTTAGTTCAACTATG-GCTACGAGT
       ::::::::: : ::::  ::::: ::::: :::: :::::::: :::: : :::::::::
Munich CCACTACTTTG-ATACAATGTAATGTGTAGAAGTAGTTAGTTCTACTA-GAGCTACGAGT
           700        710       720       730       740        750

            760                    770                          780
R1     ACAGAACTATT----------TGA---CAAT----------AGA---------ACTTCAG
       :  ::::::::          :::   : ::          :::         ::   ::
Munich AT-GAACTATTGGTTTAGGAGTGATTACTATCCGTACCATCAGATGAAAGGGTAC---AG
              760       770       780       790       800

                790
R1     --TTCCCGTCAT--TTAAG--------------------------------------TGT
         :::::: :::  :::::                                      :::
Munich ATTTCCCGACATAGTTAAGGGTAAATCATAAGTAGATTACTCTGCTACAACCATGTTTGT
       810       820       830       840       850       860

      800       810       820       830       840        850
R1     AACTGTTATTAGTGACTGTTGGGTGGTTTAGTTGTTAGGGTATTC-CATCGCA-CCTGAT
       ::::::::   ::::::::  ::: ::::::::: :: ::::::: :: :::: ::::::
Munich AACTGTTA---GTGACTGTAAGGTAGTTTAGTTGCTAAGGTATTCTCA-CGCAACCTGAT
       870          880       890       900       910        920

        860           870       880        890       900       910
R1     AGTGAT----GCGCATCACAGGAAGGGAGGA-TCCTATGAGAGGGAATAGCTTGGGCAAC
       ::::::        ::::::::::::    : :: ::  :::::::::::::: :::::
Munich AGTGATATAT----ATCACAGGAAGG----ACTCGTA--AGAGGGAATAGCTTAGGCAAT
            930          940           950         960       970
```

```
             920        930        940         950        960
R1     AACCAAGTGAACGTA-GTGAACGTAGATACCC-ACCATGCAGAACAGTATGTG------G
       :::: :::::::::: ::::::::::::::: :: :::::::::: : :::      :
Munich AACCTAGTGAACGTATGTGAACGTAGATACCCTAC-ATGCAGAACAG-A-GTGTTTGAAG
           980        990      1000        1010       1020       1030

            970        980        990      1000       1010
R1     AACAG-AGTGTTCAGGAAGCGTAGCGTTAGCGAAGCGAATGTAGTAGAACA
       ::::: :: :::::: ::::::::::::::::::::::: :::::::::
Munich AACAGGAG-GTTCAGTAAGCGTAGCGTTAGCGAAGCGAATG-AGTAGAACA
              1040       1050       1060      1070
```
